# Relationship between fatty infiltration of paraspinal muscles and clinical outcome after lumbar discectomy

**DOI:** 10.1016/j.bas.2022.101697

**Published:** 2022-12-05

**Authors:** Vasco Carvalho, Juliana Santos, Pedro Santos Silva, Rui Vaz, Paulo Pereira

**Affiliations:** a Department of Neurosurgery, Centro Hospitalar Universitário São João, Porto, Portugal; b Faculty of Medicine of the University of Porto, Portugal

**Keywords:** Fat infiltration, Paravertebral lumbar muscle, Lumbar disc herniation, Outcome, Lumbar surgery

## Abstract

**Introduction:**

Cross sectional area (CSA) and fat infiltration (FI) are important parameters to assess paravertebral muscle atrophy. However, the relationship of muscular fat infiltration in patients with symptomatic lumbar disc herniation undergoing surgery remains unclear.

**Research question:**

Does lumbar paravertebral muscle atrophy have prognostic value regarding the clinical outcome for patients with symptomatic lumbar disc herniation undergoing surgery?

**Methods:**

Patients over 18 years of age with lumbar disc herniation and radicular pain who underwent single-level discectomy were included. Multifidus, erector spinae and psoas cross-sectional area (CSA) and fatty infiltration (FI) were measured by ImageJ software at the levels of L3-L4, L4-L5 and L5-S1 from T2-weighted Magnetic Resonance axial images. Clinical status was assessed preoperatively and one-year after surgery with patient reported outcome measurements (PROMS), that included Numeric Rating Score for back and leg pain, Core Outcome Measurement Index (COMI), Oswestry Disability Index and EuroQoL-5D. Univariate and multiple linear regressions were performed.

**Results:**

Erector spinae FI was the only muscle-related factor that correlated to postoperative PROMS. Postoperative COMI was higher in patients with FI>30% (median: 4.4, IQR: 3.2) and lower when FI<15% (median: 1.2, IQR: 1.6) (Kruskal-Wallis, p ​< ​0.001). Male gender was associated with better outcome as well as erector spinae FI<15%, while FI >30% was related to worse postoperative status.

**Conclusions:**

In the current study, increased fat infiltration of erector spinae muscles correlated to less favorable clinical outcomes following lumbar discectomies.

## Highlights

• Patient outcome after spine surgery depends on multiple factors.
• Magnetic resonance as a tool for assessment of paravertebral muscles characteristics.
• Worse postoperative clinical outcome with higher erector spinae fat infiltration.
• First study describing the relationship of muscular fat infiltration and outcome after surgery.

## 1 Introduction

Lumbar disc herniation is a highly prevalent degenerative spine condition. Low back pain (LBP), leg pain (radicular pain) and movement restrictions are triggered when a protruding disc compresses a nerve root (Kalichman et al., 2017). Lumbar discectomy is the gold standard treatment for symptomatic lumbar disc herniation resistant to conservative care. Although most patients treated with surgery have favorable outcomes, a significant part of them fall short of expectations (Dewing et al., 2008; Mannion et al., 2018). Multiple factors have been shown to negatively influence clinical outcomes, such as older age, female gender, mood disorders, smoking habits, physical inactivity, patients’ expectations and longer symptom duration (Chin-Hung Chen et al., 2017; Jimenez-Almonte et al., 2020; Teichtahl et al., 2015a; Fortin et al., 2013; Furunes et al., 2018). Nevertheless, the evidence of the role of anatomical characteristics in clinical outcome is scarce (Wilson et al., 2016).

Paravertebral muscles act as structural and functional stabilizers of the spine (Storheim et al., 2017; Liu et al., 2019). Magnetic Resonance Imaging (MRI) allows to determine the cross-sectional area (CSA) as well as fatty infiltration (FI) of these muscles, which are considered the best parameters to assess muscle atrophy (Ogon et al., 2019; Urrutia et al., 2018). Fat infiltration reduces contractile and functional capacity of muscles (Shahidi et al., 2017; He et al., 2020; Teichtahl et al., 2015b) and higher atrophy levels of the paravertebral muscles were reported in symptomatic patients with LBP when compared to asymptomatic groups (Kalichman et al., 2017; Park et al., 2018). Several studies suggest that fatty infiltration is higher in women, increases with aging and seems to be more pronounced on the lower spine levels suggesting a craniocaudal distribution (Shahidi et al., 2017; Lee et al., 2017; Crawford et al., 2016). However, the role of muscle atrophy in the outcome of patients with symptomatic lumbar disc herniation undergoing surgery remains controversial (Arts et al., 2011).

The aim of this study is to investigate the association between muscle atrophy on preoperative MRI and clinical outcomes one year after lumbar discectomy, in order to understand the prognostic value of paravertebral muscle parameters in clinical outcome.

## 2 Materials and methods

A retrospective analysis of prospectively collected data was conducted, selecting all patients over eighteen years old with lumbar disc herniation and radicular pain who underwent single-level microdiscectomy at our department from January 2016 to December 2019. Study protocol and investigation were approved by the hospital's ethics committee. Informed consent was obtained from included patients.

Patients with deformity, spondylolisthesis, spinal stenosis, fractures, tumors and infections or previous lumbar surgeries were excluded.

**Fig. 1:**
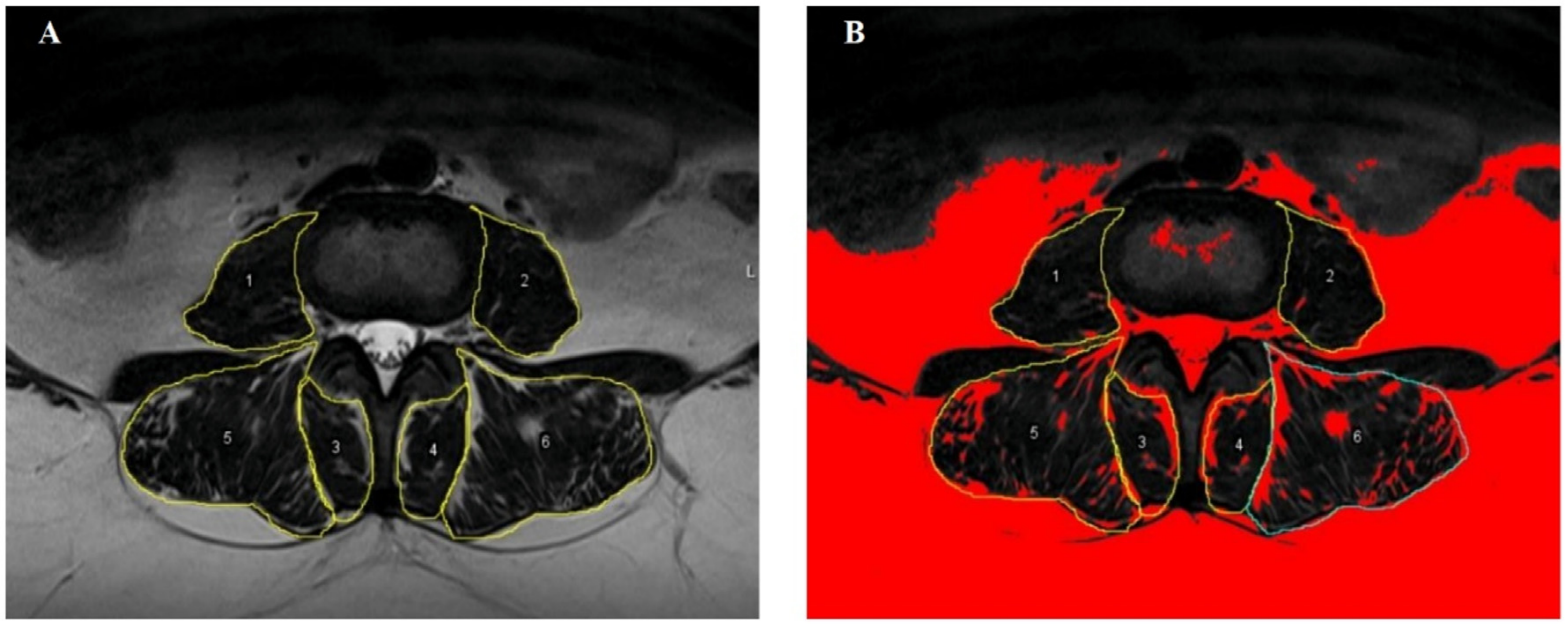
Regions of interest (ROI) (A) and fat infiltration (B) of paravertebral muscles measured by ImageJ software pseudo-colouring method, applying automatic thresholds.

All patients underwent lumbar spine MRI before surgery. Axial T2-weighted MRI slices at the levels of L3-L4, L4-L5 and L5-S1 intervertebral discs were selected. In each level, paravertebral muscles (multifidus, erector spinae and psoas) were assessed bilaterally, leading to 36 measurements for each patient. ImageJ software 1.8.0_112 version (National Institutes of Health, Bethesda, USA) was used to define each CSA by manually delimiting muscular edges of multifidus, erector spinae and psoas according to the method proposed by Crawford et al. (2017). The percentage of FI of paravertebral muscles was calculated by ImageJ software pseudocoloring method, applying automatic thresholds (Fig. 1). In order to summarize these data, median values of CSA and FI per muscle were obtained, grouping level and side as a single measure.

Clinical status was assessed according to preoperative and one-year postoperative patient reported outcome measurements (PROMS) questionnaires that included Pain Numeric Rating Scale (NRS) for back and leg pain, Core Outcome Measurement Index (COMI), Oswestry Disability Index (ODI) and EuroQoL-5D (EQ-5D).

Patients’ data were obtained from clinical records and included age, gender, weight (kg), height (cm), body mass index (kg/m^2^), smoking status, antidepressant medication and rehabilitation prior to surgery. Surgical data were also collected, such as surgery date, lumbar level of discectomy and side.

### 2.1 Statistical analysis

R software (R Foundation for Statistical Computing, Vienna, Austria) version 4.0.3 was used for data analysis. Sample size calculation was performed considering postoperative COMI as the primary end point, leading to COMI mean comparison between two groups according to muscle characteristics (CSA or FI). The calculated sample size was 98, for one sided T-test, significance level of 0.05, power of 0.95 and effect size of 0.67. Effect size was calculated according to Cohen (1988): for means difference we used 1.7, the minimal detectable change (MDC) for Portuguese language COMI (Damasceno et al., 2012), and we considered the standard deviation (SD) of 2.7 for postoperative COMI in patients with disc herniation (DH) from the study by Mannion et al. (2018).

Spearman's correlation (rs) was used for correlation between continuous variables; Kruskal-Wallis or Wilcoxon tests for mean comparison between independent or dependent samples, Fisher's test was used for associations between categorical variables.

Multiple linear regression was performed for adjustment for other variables. Decisions about the independent variables to include in the model were done by univariable selection with simple linear regression, based in a threshold of 0.1 for p-value, and then with backward selection. Continuous variables with non-linear relations to outcome were split in categories, based on visual plot analysis (locally estimated scatterplot smoothing) and in a step function (Supplemental file).

## 3 Results

**Fig. 2:**
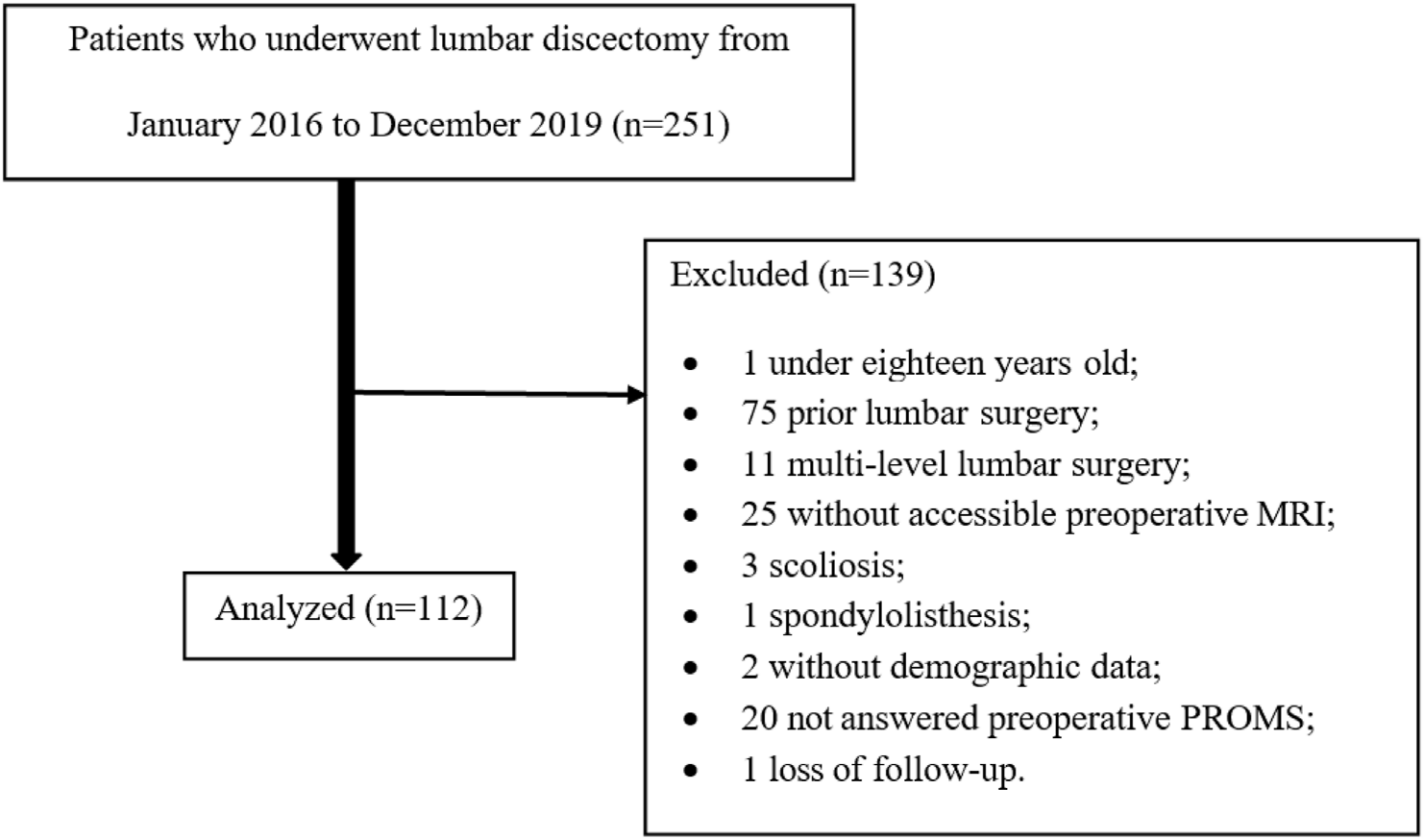
Flow diagram of participant screening, exclusion, and analysis.

From 251 patients screened for eligibility, 112 patients (59 female and 53 male) were included in this study (Fig. 2). The mean age was 46.1 ​± ​12.7 years, ranging between 20 and 78 years old. The average BMI was in the overweight category (27.1 ​± ​3.89 ​kg/m^2^). Lumbar discectomies were performed at levels L2-L3 (1.8%), L3-L4 (4.5%), L4-L5 (48.2%) and L5-S1 (45.5%). Patient characteristics are presented in Table 1.

**Table 1:** Patient demographic and paraspinal muscle characteristics.

	Overall (N ​= ​112)
Gender	
Female	59 (52.7%)
Male	53 (47.3%)
**Age, mean (SD)**	46.1 (12.7)
**BMI, mean (SD)**	27.1 (3.9)
**Rehabilitation prior to surgery**	
No	25 (22.3%)
Yes	87 (77.7%)
**Smoker**	
No	84 (75.0%)
Yes	28 (25.0%)
**Antidepressant drugs**	
No	64 (57.1%)
Yes	48 (42.9%)
**Level**	
L2-L3	2 (1.8%)
L3-L4	5 (4.5%)
L4-L5	54 (48.2%)
L5-S1	51 (45.5%)
**Pain Side**	
Left	60 (53.6%)
Right	45 (40.2%)
Bilateral	7 (6.2%)
**Preoperative PROMS, median (IQR)**	
COMI	7.8 (6.4, 9.0)
ODI	46.0 (34.8, 62.0)
NRS back	7.0 (5.0, 8.0)
NRS leg	8.0 (6.0, 9.3)
EQ-5D	0.516 (−0.009, 0.620)
**Postoperative PROMS, median (IQR)**	
COMI	3.2 (1.5, 4.9)
ODI	22.0 (12.0, 36.0)
NRS back	3.0 (1.0, 5.3)
NRS leg	3.0 (1.8, 5.0)
EQ-5D	0.795 (0.648, 0.879)
**CSA, median (IQR)**	
Erector spinae	3025.250 (1844.875, 3955.125)
Multifidus	1702.500 (1175.625, 2495.625)
Psoas	195.250 (119.125, 313.625)
**Fat Infiltration %, median (IQR)**	
Erector spinae	26.517 (20.816, 31.861)
Multifidus	19.191 (13.219, 24.536)
Psoas	1.977 (1.275, 2.945)

**Fig. 3:**
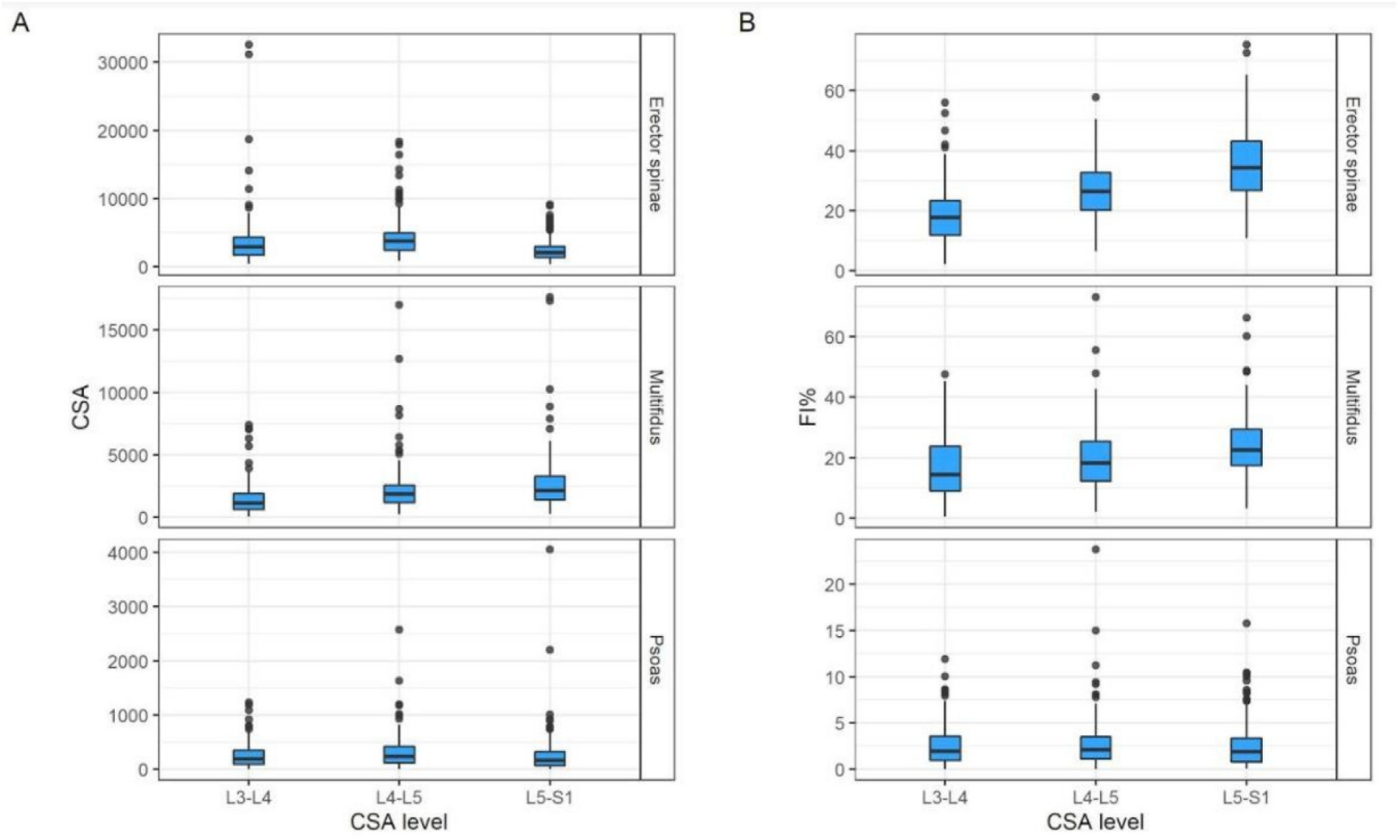
Cross sectional area (CSA) (A) and fat infiltration (FI) percentage (B) at each disc level for erector spinae, multifidus and psoas muscles.

Regarding the morphology of paravertebral muscles on MRI, erector spinae had the highest median cross section area (CSA) values (3025; IQR: 1845–3955), followed by multifidus (1703; IQR: 1176–2497) and psoas (195; IQR: 119–314). For erector spinae the median fat infiltration (FI) was also higher (27%; IQR: 21–33%) than for multifidus (19%; IQR: 13–25%) and psoas (2%; IQR: 1–3%) (Table 1). Fat infiltration percentage increased slightly from L3-L4 to L5-S1 on erector spinae and multifidus muscles. Psoas muscle CSA and FI remained stable across all spinal levels (Fig. 3). For each muscle, no significant differences were found between sides for CSA or FI (Wilcoxon tests, p values between 0.12 and 0.85).

### 3.1 MRI analysis and preoperative clinical assessment

Psoas muscle CSA expressed weak to moderate correlations with preoperative PROMS, specifically with COMI (rs ​= ​−0.29), ODI (rs ​= ​−0.32) and EQ5D(rs ​= ​0.28) scores. Both the CSA and FI of multifidus and erector spinae presented no correlation with patient clinical status before surgery.

### 3.2 MRI analysis and postoperative clinical assessment

Erector spinae FI was the only muscle-related factor that correlated to postoperative PROMS. In particular, erector spinae FI had a moderate correlation with postoperative COMI (rs ​= ​0.38), leg NRS (rs ​= ​0.39) and EQ5D (rs ​= ​−0.3); and weak correlations with postoperative ODI and NRS-back (rs ​= ​0.26 and 0.24).

**Fig. 4:**
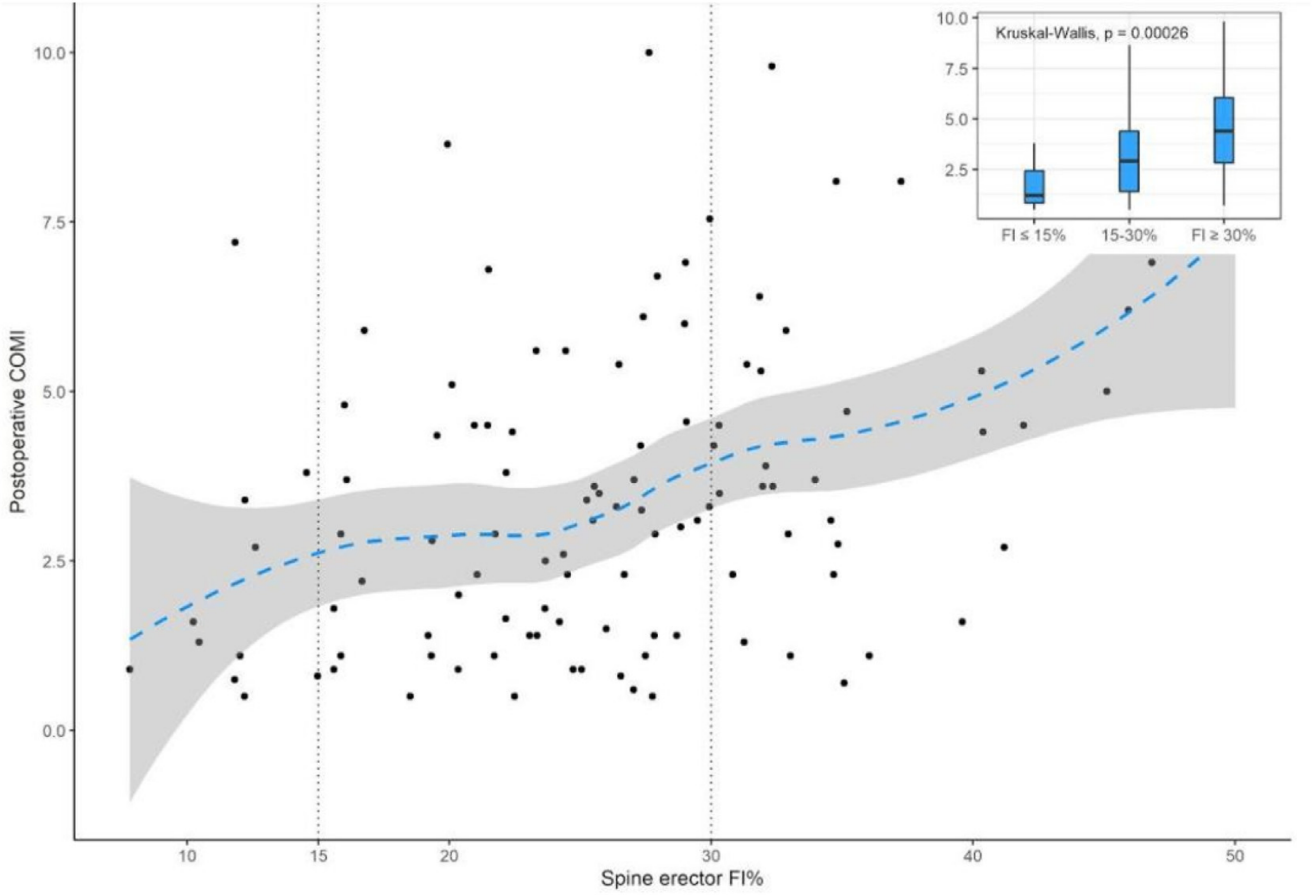
Relation between erector spinae FI and postoperative COMI. Boxplots: postoperative COMI according to erector spinae FI groups.

In order to confirm erector spinae FI as an independent factor related to postoperative COMI, a multiple linear regression model was performed to control confounding variables. Univariable simple linear regression was used and identified gender (p ​< ​0.001), age (p ​= ​0.02) and height (p ​= ​0.01) as being associated with postoperative COMI (supplemental file). As the relation between erector spinae FI and postoperative COMI was not linear (Fig. 4), that variable was split in three groups: FI < 15% (10% of patients), FI 15–30% (59% of patients), FI ​> ​30% (31% of patients). From clinical and statistical perspective, postoperative COMI was significantly different according to erector spinae FI groups (boxplots – Fig. 4): postoperative COMI was higher in FI > 30% group (median: 4.4, IQR: 3.2) and lower in FI ​< ​15% (median: 1.2, IQR: 1.6) (Kruskal-Wallis, p ​< ​0.001). Table 2 summarizes baseline characteristics of the patients included in each of the three groups.

**Table 2:** Baseline characteristics of the patients included in each of the three groups for FI of erector spinae.

	**FI ​≤ ​15% (N ​= ​11)**	**15**–**30% (N ​= ​66)**	**FI ​≥ ​30% (N ​= ​35)**	**p value**
**Gender**				0.222^a^
**Female**	3 (27.3%)	36 (54.5%)	20 (57.1%)	
**Male**	8 (72.7%)	30 (45.5%)	15 (42.9%)	
**Age, median (IQR)**	35.0 (30.5,42.0)	45.5 (36.0,50.8)	50.0 (43.0,63.0)	<0.001^b^
**BMI, median (IQR)**	24.7 (23.7,27.8)	26.3 (24.3,28.1)	28.4 (25.3,32.2)	0.019^b^
**Rehabilitation prior to surgery**				0.157^a^
**No**	5 (45.5%)	14 (21.2%)	6 (17.1%)	
**Yes**	6 (54.5%)	52 (78.8%)	29 (82.9%)	
**Smoker**				0.256^a^
**No**	6 (54.5%)	51 (77.3%)	27 (77.1%)	
**Yes**	5 (45.5%)	15 (22.7%)	8 (22.9%)	
**Antidepressant drugs**				0.538^a^
**No**	8 (72.7%)	37 (56.1%)	19 (54.3%)	
**Yes**	3 (27.3%)	29 (43.9%)	16 (45.7%)	
**Preoperative PROMS, median (IQR)**				
**COMI**	7.8 (6.5, 9.0)	7.6 (6.5, 9.0)	7.9 (6.4, 9.1)	0.806^b^
**ODI**	48.0 (25.0, 68.0)	46.0 (38.0, 60.0)	46.0 (35.0, 59.0)	0.997^b^
**NRS back**	7.0 (4.5, 8.5)	7.0 (5.0, 8.0)	7.0 (5.0, 9.0)	0.991^b^
**NRS leg**	6.0 (4.0, 8.5)	8.0 (6.0, 9.0)	8.0 (6.5, 10.0)	0.303^b^
**EQ-5D**	0.516 (0.353,0.603)	0.364 (−0.016,0.620)	0.516 (0.055, 0.587)	0.616^b^
**Postoperative PROMS, median (IQR)**				
**COMI**	1.3 (0.9, 3.1)	2.9 (1.4, 4.5)	4.4 (2.8, 6.1)	0.002^b^
**ODI**	22.0 (10.0, 24.0)	19.0 (12.0, 32.0)	28.0 (17.0, 40.0)	0.064^b^
**NRS back**	2.0 (0.0, 3.5)	3.0 (2.0, 4.8)	5.0 (1.0, 6.0)	0.117^b^
**NRS leg**	2.0 (0.0, 3.5)	2.0 (1.0, 4.8)	5.0 (3.0, 7.0)	0.001^b^
**EQ-5D**	0.837 (0.837,1.000)	0.782 (0.682,0.879)	0.727 (0.592, 0.837)	0.032^b^
**CSA,median (IQR)**				
**Erector spinae**	1251.5(1151.5,2368.8)	3039.0(1816.8,3940.4)	3219.5 (2635.8,4635.8)	0.002^b^
**Multífidus**	1010.5(637.5,1702.3)	1751.0(1116.0,2628.8)	1777.0 (1449.0,2797.3)	0.020^b^
**Psoas**	144.0 (96.0,380.3)	187.0 (117.4274.0)	244.0 (136.0,338.0)	0.237^b^
**Fat infiltration median (IQR)**				
**Erector spinae**	12.0 (11.1, 12.4)	24.3 (20.5, 27.2)	34.7 (32.0, 40.0)	< 0.001^b^
**Multífidus**	10.3 (8.5, 12.5)	17.31 (13.2, 22.7)	17.3 (13.2, 22.7)	< 0.001^b^
**Psoas**	1.1 (0.7, 1.7)	1.8 (1.1, 2.8)	2.6 (1.8, 4.4)	< 0.001^b^

a Fisher's Exact Test for Count Data.

b Kruskal-Wallis rank sum test.

In multiple linear regression model (Table 3), erector spinae FI and gender were the only independent factors related to postoperative status measured by COMI. Male gender was associated with better outcome as well as erector spinae FI < 15%, while FI > 30% was related to worse postoperative status.

**Table 3:** Multivariable regression model for postoperative COMI.

Characteristic	Beta	95% CI	p-value
(Intercept)	2.5	1.2, 3.9	<0.001
**Fat infiltration of erector spinae**			
≤ ​15%	–	–	
15–30%	1.1	−0.28, 2.4	0.12
≥30%	2.4	1.0, 3.8	0.001
**Gender**			
Female	–	–	
Male	−1.1	−1.8, −0.29	0.007

## 4 Discussion

Integrity of the paravertebral muscles (multifidus, erector spinae and psoas) ensures the normal function and alignment of the spine (He et al., 2020); this musculature contains a high proportion of low tonic and fatigue-resistant fibers (type I), reflecting their role in maintaining posture and joint stability (Kalichman et al., 2017). On the other hand, fat infiltration of these muscles is a sign of atrophy and has been associated with functional impairment, spine instability and low back pain (Teichtahl et al., 2015b). Physical inactivity has also been correlated with atrophy and morphological abnormalities in the lumbar spine, which results in low back pain and disability (Teichtahl et al., 2015a).

Lumbar disc herniation may cause nerve roots impairment, which leads to structural changes in paravertebral muscles, such as increased fatty infiltration, atrophy of type I fibers and conversion to type II fibers (Kalichman et al., 2017), which can be related to low back pain and poorer spine function. In the current study, fat infiltration of the multifidus and erector spinae muscles revealed no correlation to preoperative PROMS. This result is in line with a retrospective study, conducted by Bhadressha et al. (Bhadresha et al., 2016), including 165 patients with lumbar disc herniation, which concluded that muscle atrophy and fat content of lumbar paravertebral muscles were not associated with PROMS. Similar findings were reported by Hildebrandt et al. who found no significant correlation between fat infiltration of the multifidus muscles and self-assessed functional disability (Hildebrandt et al., 2017). In the present study, we found weak to moderate correlations between psoas muscle CSA and preoperative PROMS. Barker et al. also found a positive correlation between the decrease in CSA of the psoas on the affected side and the pain scores (Barker et al., 2004), although the results in literature are contradictory, as patients with degenerative lumbar spine changes also had bigger CSA of psoas muscles compared to asymptomatic controls (Arbanas et al., 2013).

In the present study, we performed a multilevel spine muscles evaluation that demonstrated a tendency of cranial to caudal increase of FI on erector spinae and multifidus. Several other studies found the exact same results, in which fat infiltration was far more evident on the lower lumbar spine levels (Kalichman et al., 2017; Storheim et al., 2017; Urrutia et al., 2018; Bhadresha et al., 2016). Hence, as stressed by Urrutia et al. no single-level muscle features are representative of the whole lumbar spine (Urrutia et al., 2018).

The literature raises the hypothesis that paravertebral muscle atrophy and higher levels of fat infiltration are related to less favorable clinical outcome after lumbar discectomy surgery, even though scientific evidence is still very limited. Storheim et al. found, in a randomized study with 173 patients submitted to a total disc replacement, that a lower multifidus fat infiltration ratio predicted a better clinical outcome (Storheim et al., 2017). Similar results were reported by Yang Liu et al. and Zotti and al., who concluded that less preoperative fat infiltration of the multifidus muscles is a predictor of better clinical outcome in patients with lumbar spinal stenosis (Liu et al., 2019; Zotti et al., 2017). Our study found no correlation between multifidus CSA and postoperative outcomes. However, lower rates of fat infiltration of the erector spinae muscles were related to better postoperative clinical outcomes. All these results together support the hypothesis that muscle atrophy and especially higher levels of fat infiltration are related to poor prognosis and could be an effective prognostic measure of postoperative outcomes. However, unlike the present research, most of the referred studies did not follow either a standard quantitative or semi-quantitative calculation for ROI, did not specify the muscular groups and did not include pre and postoperative patient outcome measures.

Regarding age and gender factors, our results are in line with previous reported results. Our analysis indicated that older patients presented higher fat infiltration rates, which supports the physiopathological evidence that the aging process decreases skeletal muscle mass and promotes its replacement by noncontractile connective tissue (Shahidi et al., 2017; Lee et al., 2017; Bhadresha et al., 2016; Tamai et al., 2018; Colakoglu and Alis, 2019; Kjaer et al., 2007). In accordance to former reports, we did not find any effect of BMI on paravertebral muscle atrophy (Fortin et al., 2013; Lee et al., 2020). It is possible that higher BMI values, by themselves do not translate into an increase in adipose infiltration at the paravertebral muscles. Smoking habits, antidepressant medication and physical therapy prior to surgery, none of them seemed to correlate with paravertebral atrophy, nor influence postoperative outcome. A surprisingly high rate of antidepressant drugs was found in our population with almost 43% of the patients taking at least one class of medication. This did not prove to be correlated in any of our study groups. Given the nature of our study, we could not address this as a direct link to depression and/or evaluate any clinical scores. Moreover, it is possible that this should probably be an overestimation of the patients with actual depression, as many antidepressant drug classes may have other indications for use, including chronic pain management.

This study emphasizes that multiple factors are likely to influence postoperative outcomes after lumbar discectomy. Patients with worse paravertebral musculature, higher percentage of fatty infiltration of erector spinae muscles and female patients presented poorer clinical outcomes. This knowledge may guide our practice and help encouraging patients to reduce inactivity and implement supervised and specific training to improve their functionality, muscle-mass and hopefully reach better postoperative outcomes (He et al., 2020). Few studies have suggested that specific training can reduce symptoms and reverse the degenerative process in paravertebral muscles. Kim et al. (2014) reported positive results after eight weeks of training in patients with degenerative disc disease. A case-control study, by Storheim et al. (2017) revealed a tendency to increase muscle CSA and density in the group of patients who practiced a biweekly exercise protocol for 15 weeks. It is important to stress however that the inefficacy of conservative treatment or inability to obtain optimal conditions for surgery should not be a reason for withdrawing the indication for surgery. Cases where further optimization of preoperative conditions is not possible require an individualized assessment but should not refrain the surgeon from proceeding with the correct indication.

Some limitations can be identified in this study. It is a single-center study with a retrospective design, which limits the generalizability of the results. Surgeries were performed by different surgeons; therefore, it is not possible to appraise whether technical variations can impact the outcomes, in spite that most surgeries were performed by a small number of spine-specialized neurosurgeons using a similar surgical technique in the same operative room. There is also the possibility for other potential confounders, such as further medical comorbidities not taken into analysis. Although the sample size is modest (n ​= ​112), this study is adequately powered to demonstrate associations between paravertebral fat infiltration and clinical outcome. Despite having a semiautomatic method for image assessment and therefore with limited variability, image workup and cross-sectional area measurements were performed by a single observer. Validated and widely used patient-reported outcome scores were selected and a quantitative method was applied to assess the CSA and fat infiltration of the paravertebral muscles instead of a visual semi-quantitative method. Nonetheless, outlier values were assessed and reviewed with a second observer of the investigation team.

## 5 Conclusion

To our knowledge, this is the first study to demonstrate an association between erector spinae muscles anatomy on preoperative MRI and postoperative clinical outcome in patients with symptomatic lumbar disc herniations. Increased fat infiltration of erector spinae muscles correlated to less favorable clinical outcomes following lumbar discectomy.

## IRB approval/research ethics committee

Full-protocol approval.

## Declaration of competing interest

The authors declare that they have no known competing financial interests or personal relationships that could have appeared to influence the work reported in this paper.
