# Supplementary figures and images for: Relationship between fatty infiltration of paraspinal muscles and clinical outcome after lumbar discectomy
Source: Brain Spine. 2022 Dec 5;2:101697. doi: 10.1016/j.bas.2022.101697 (PMC9808467; doi:10.1016/j.bas.2022.101697)

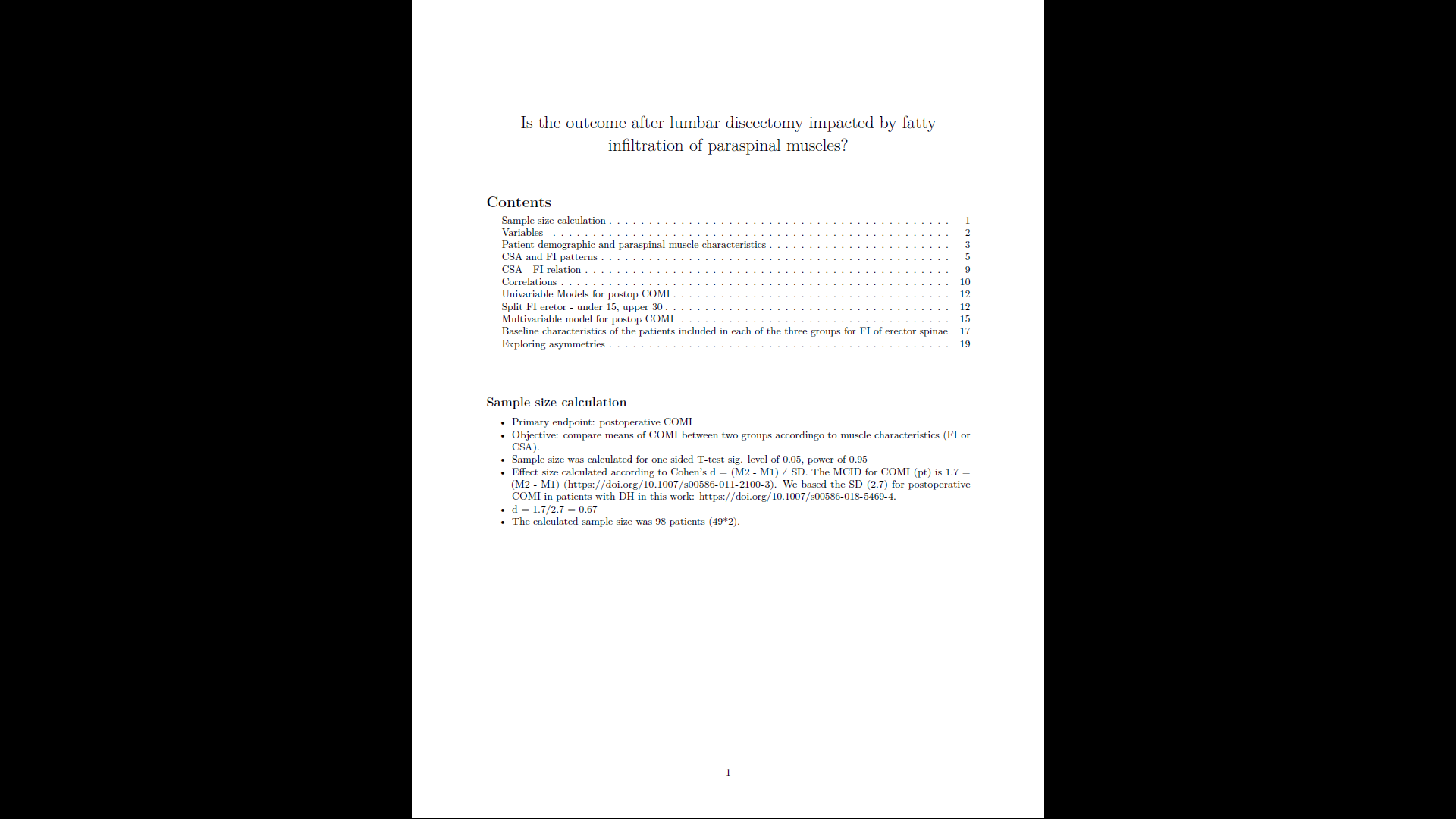

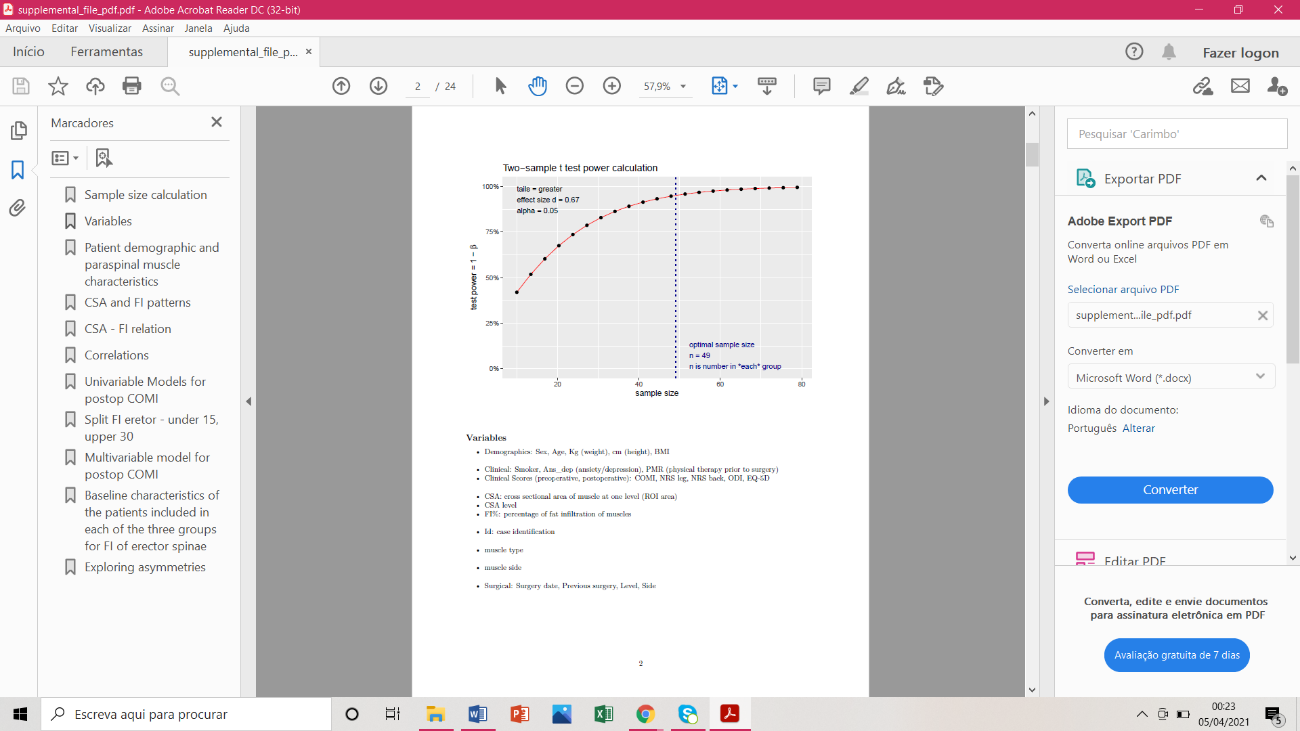


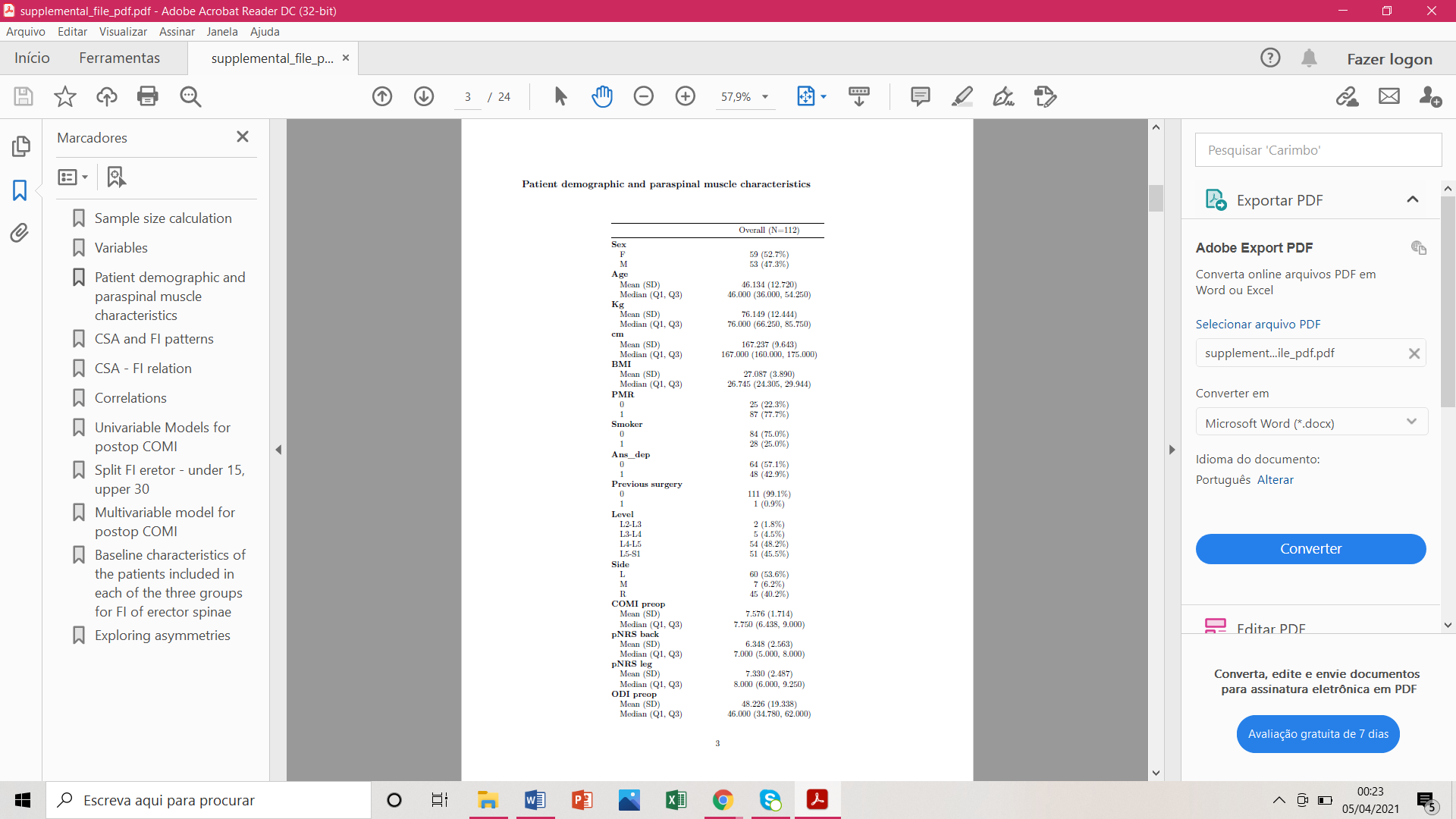


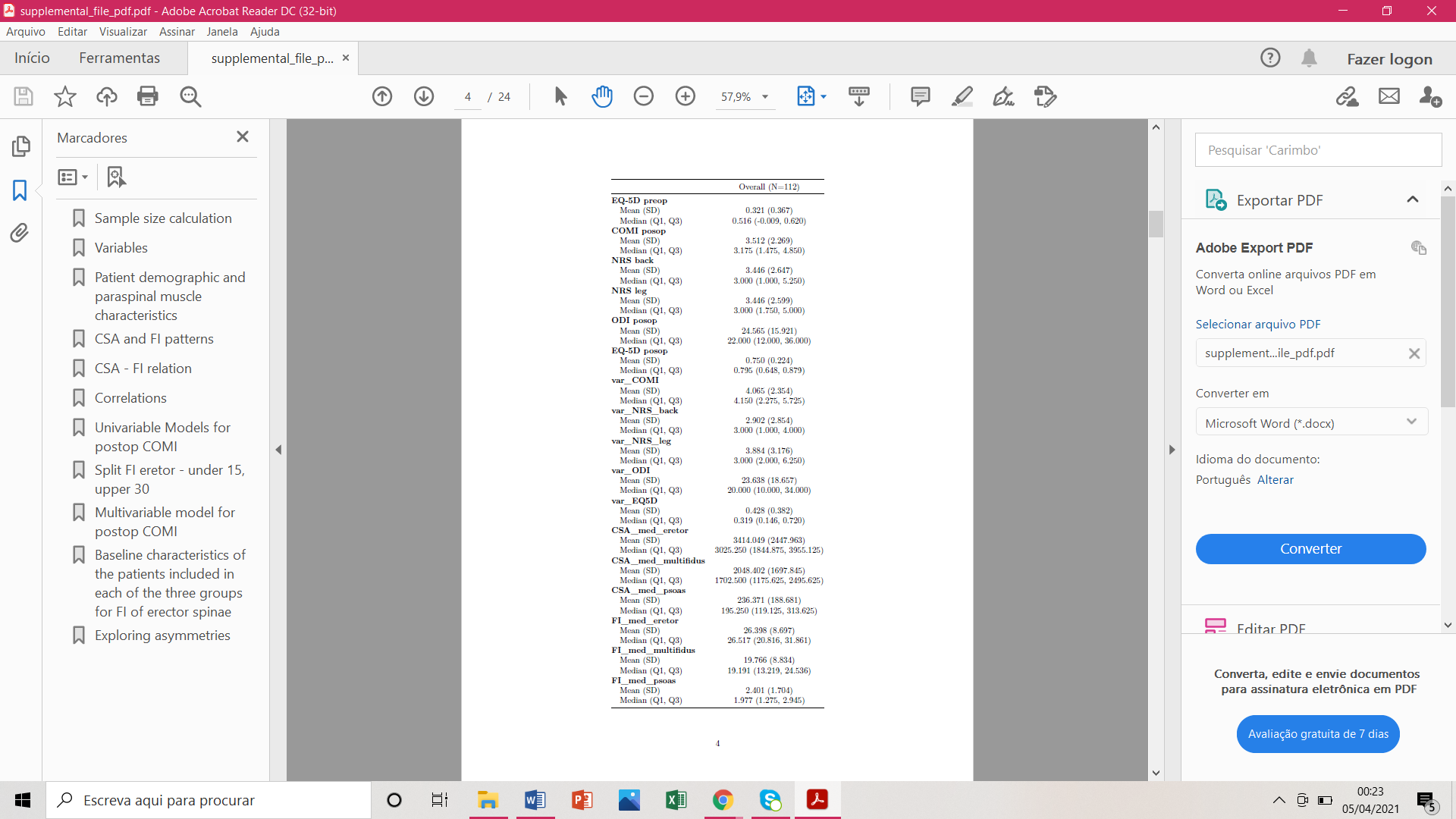


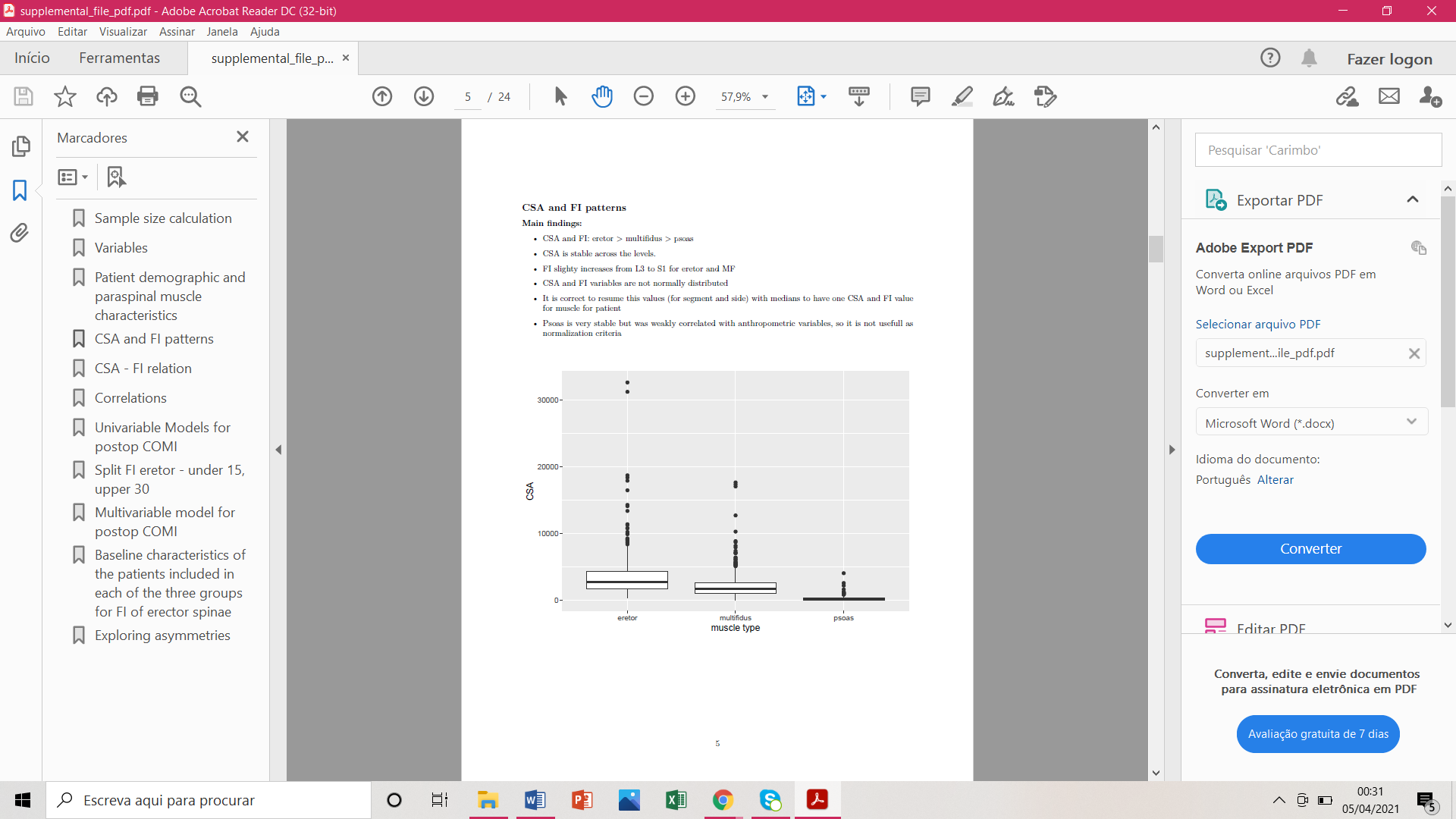


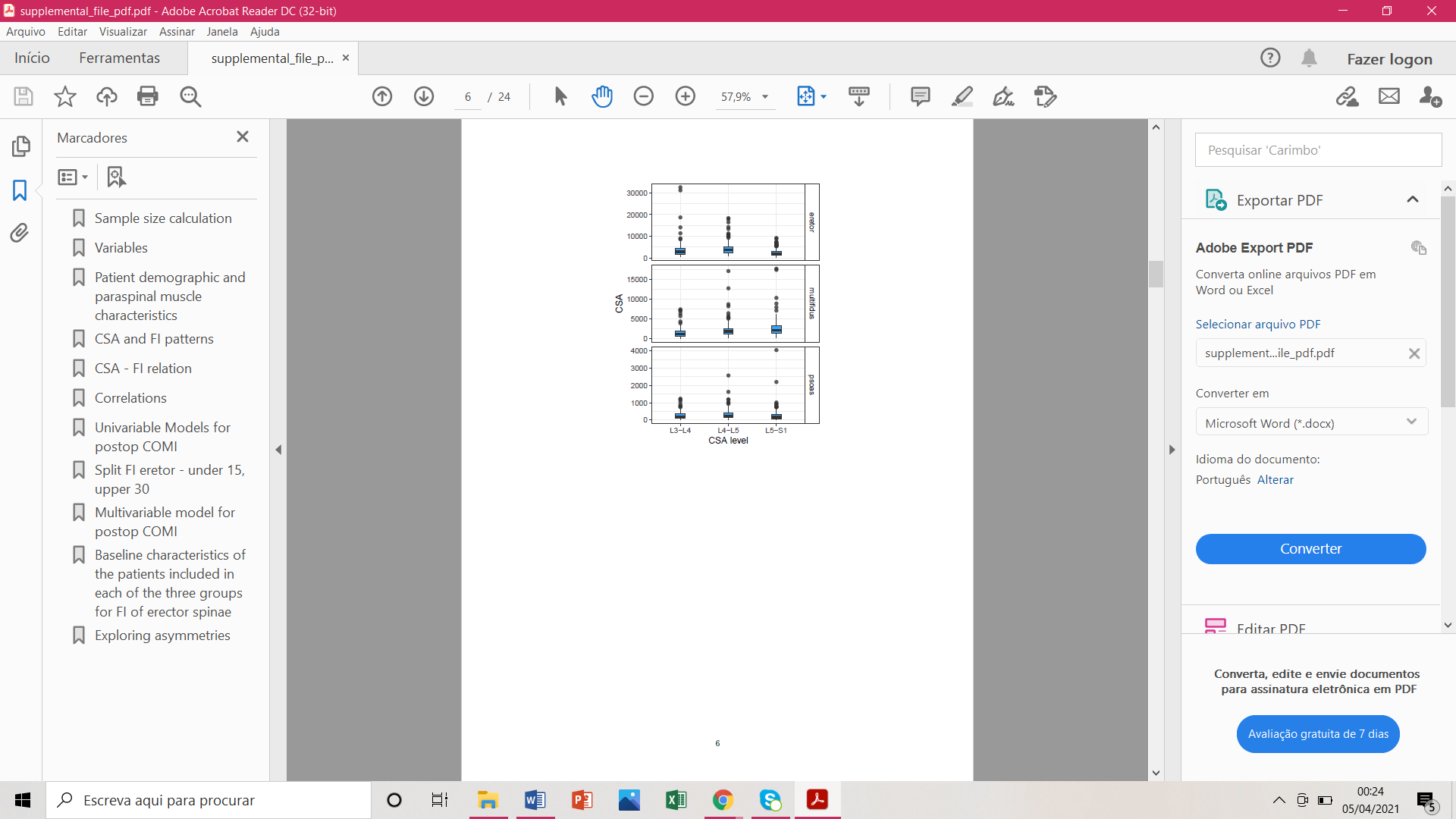


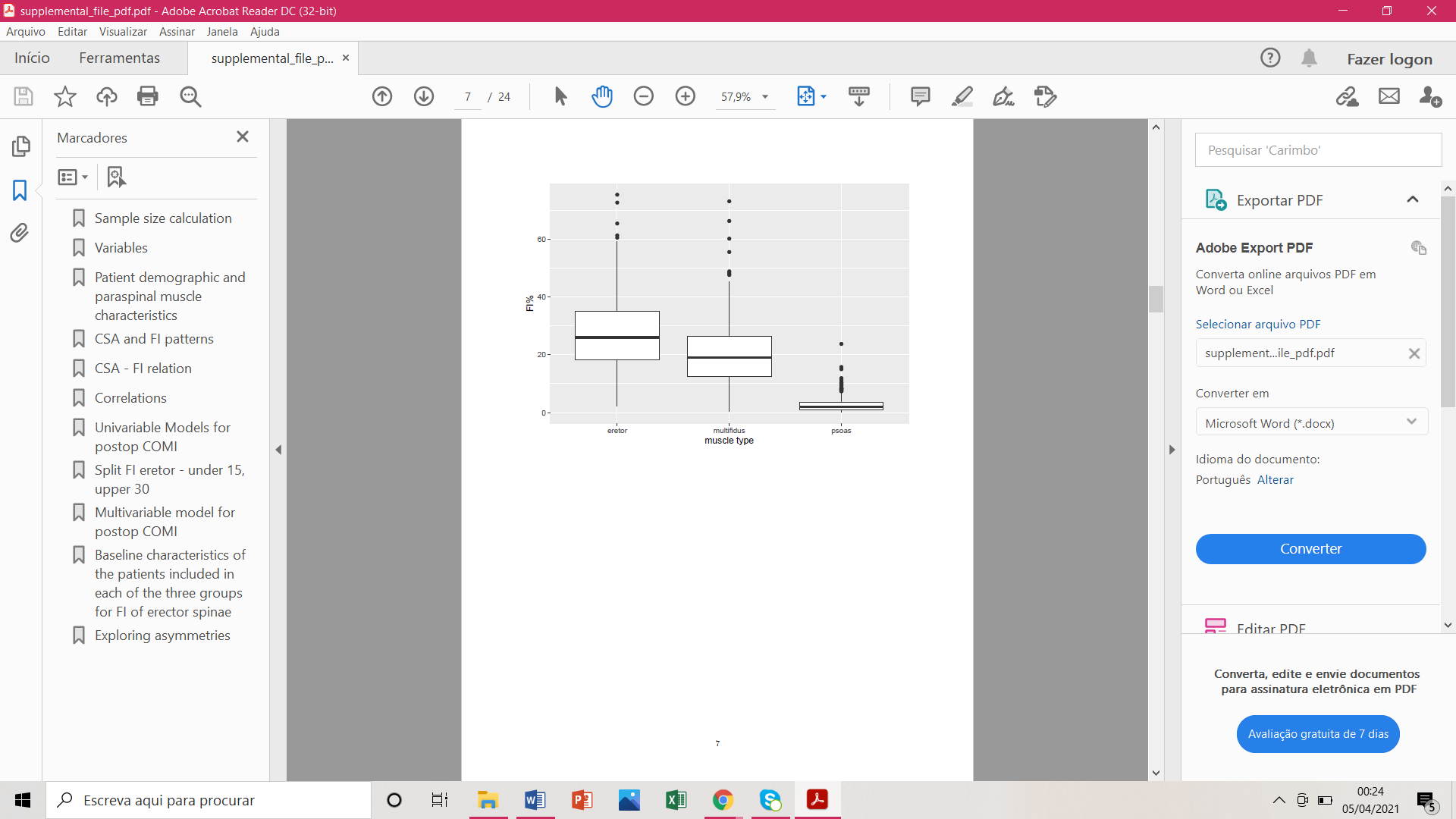


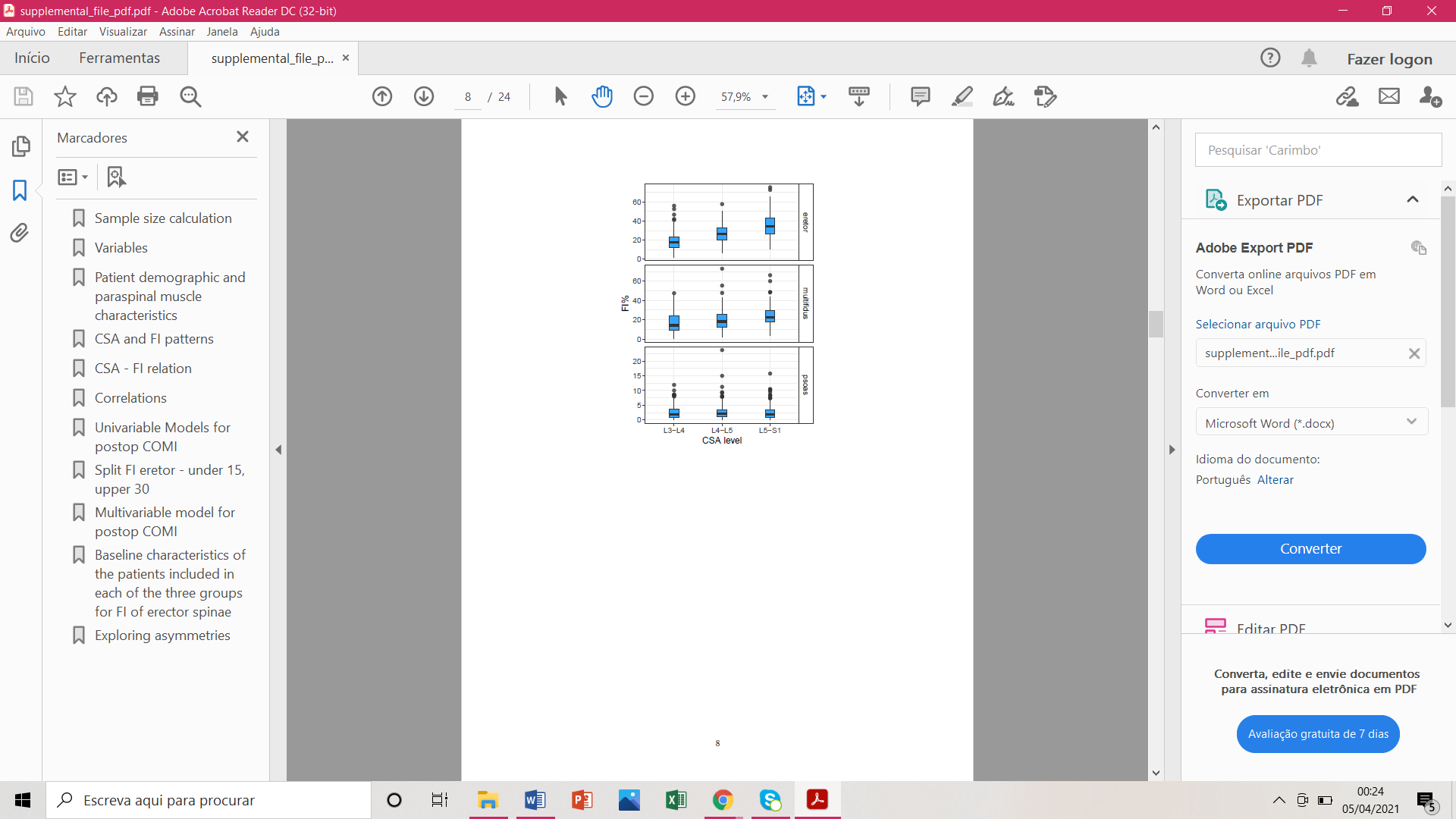


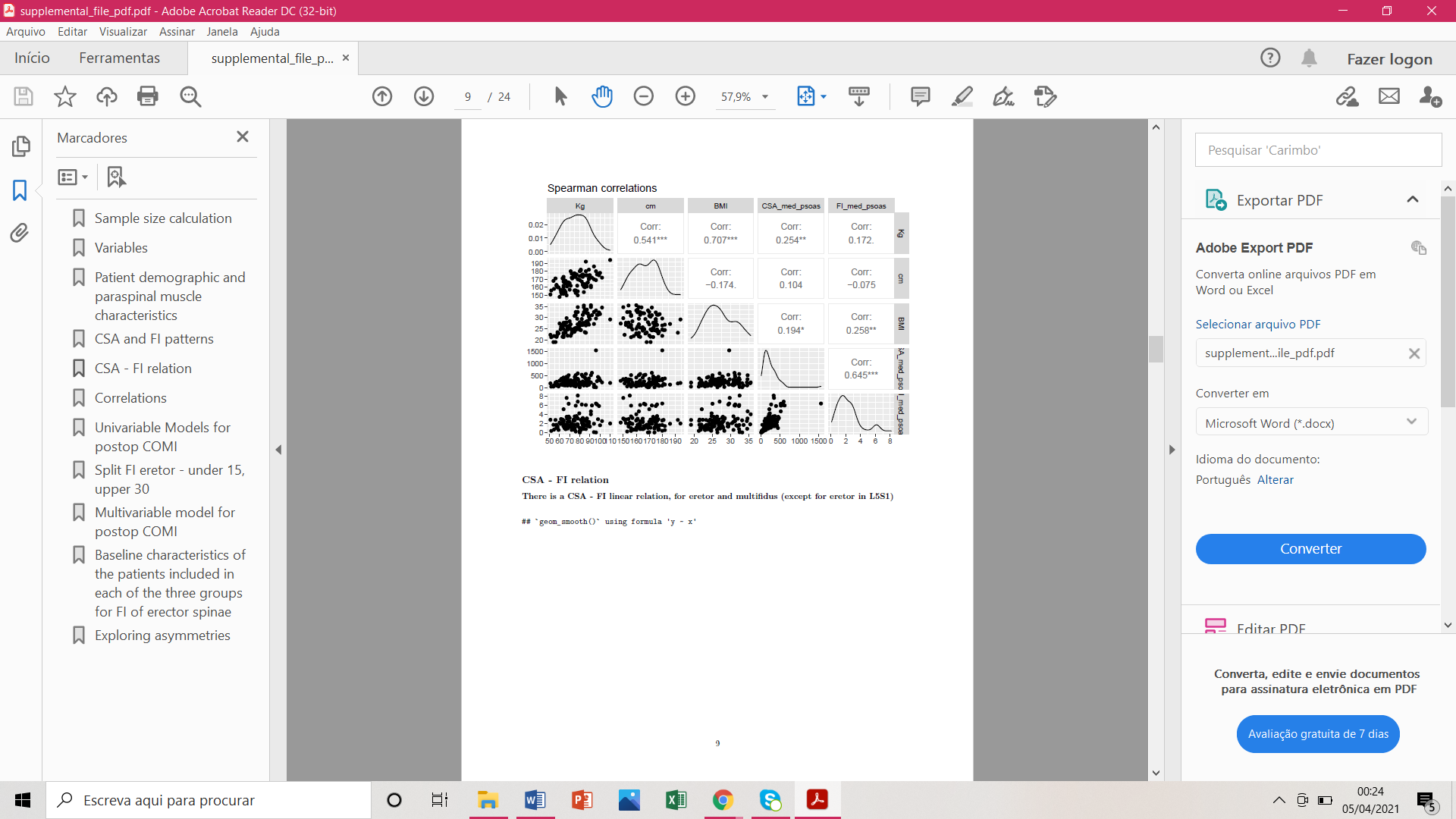


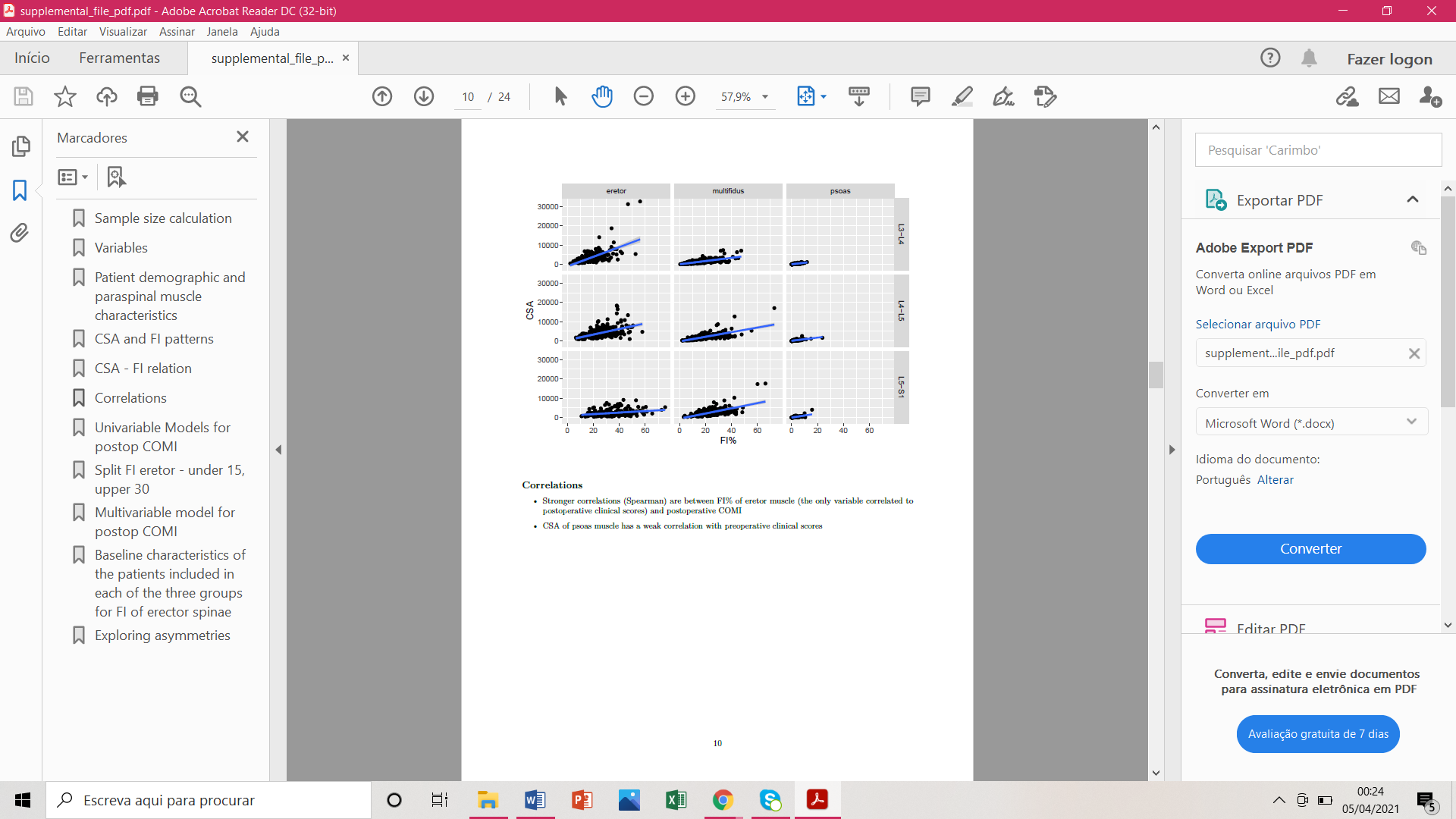


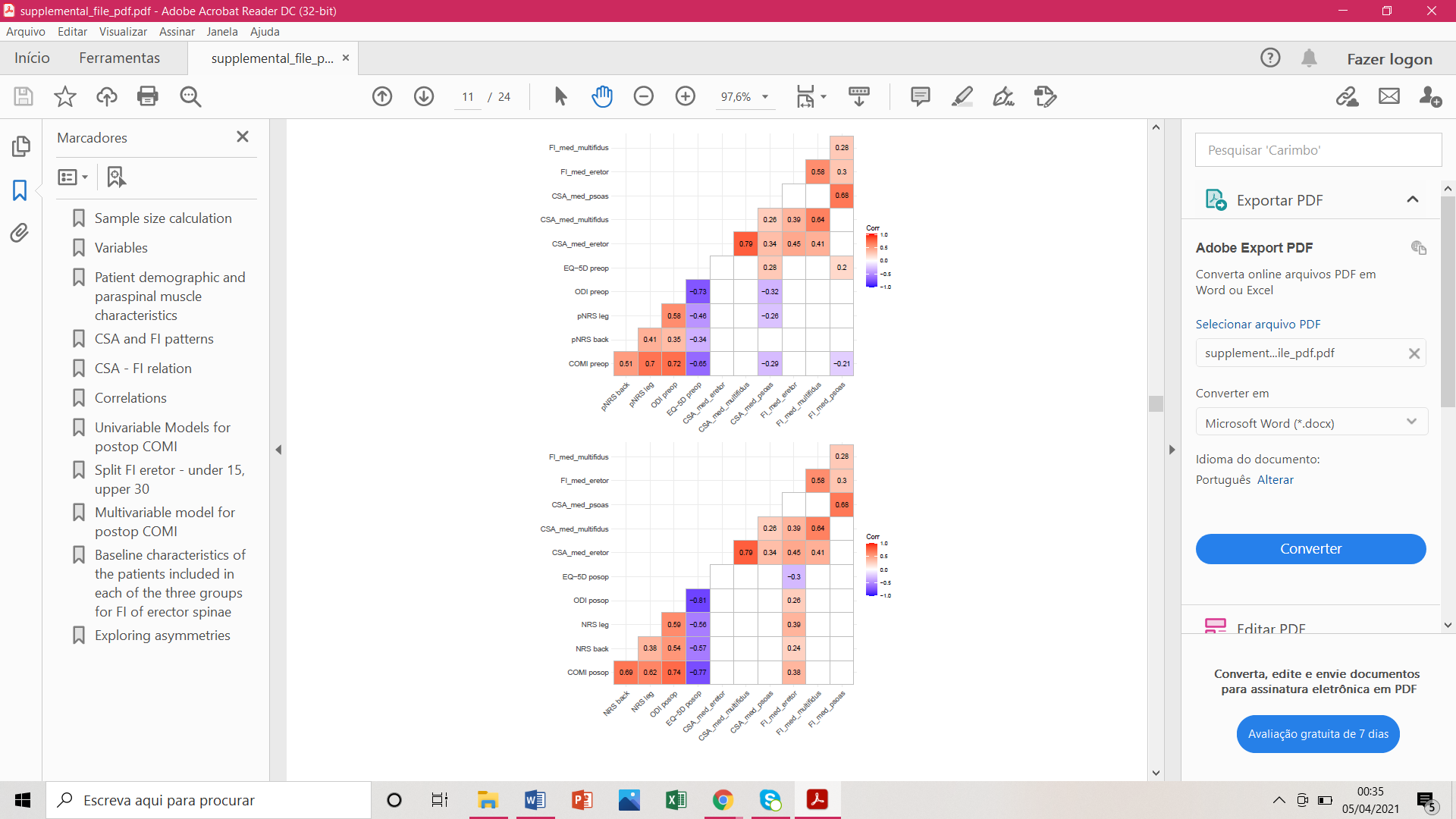


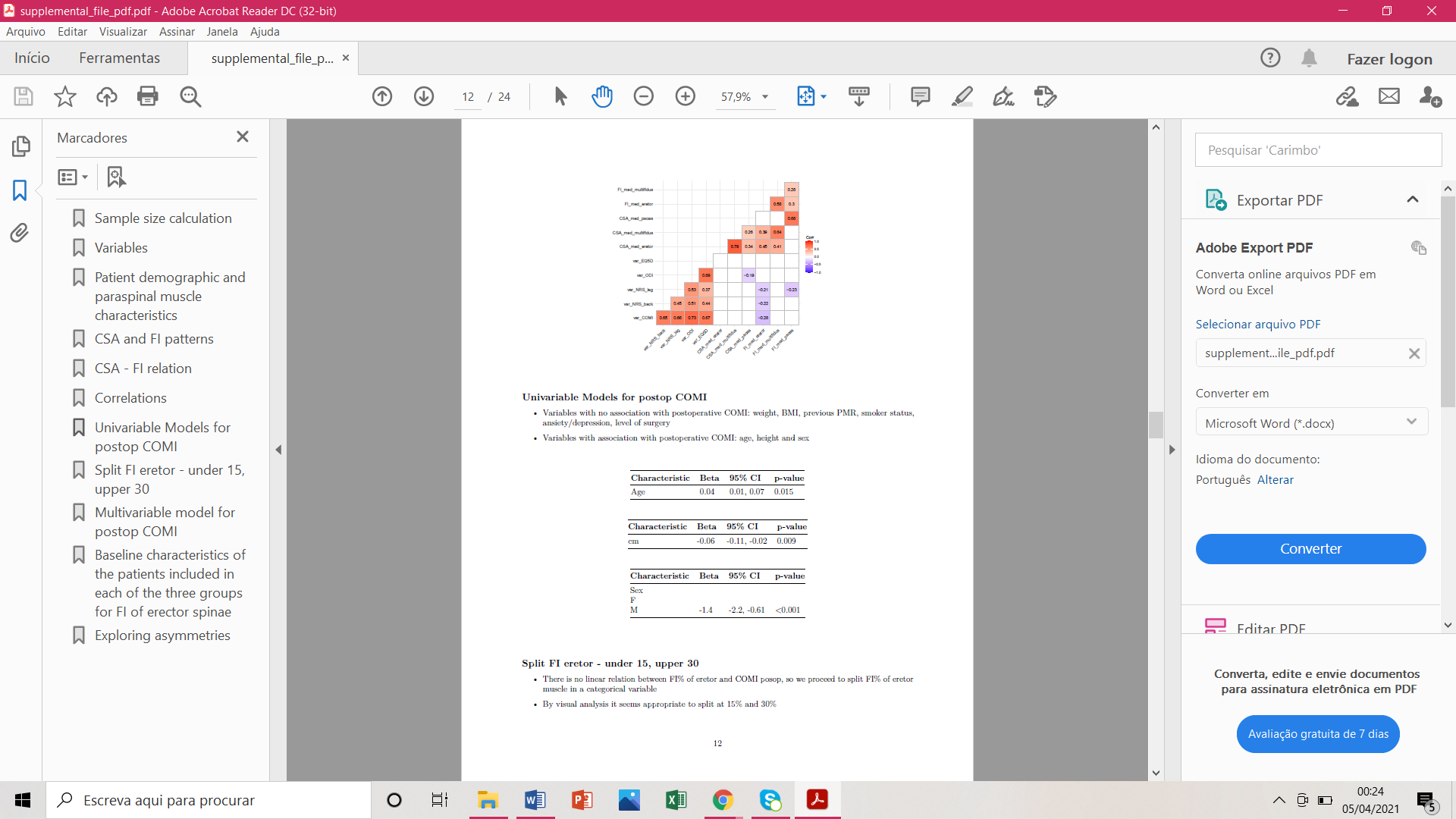


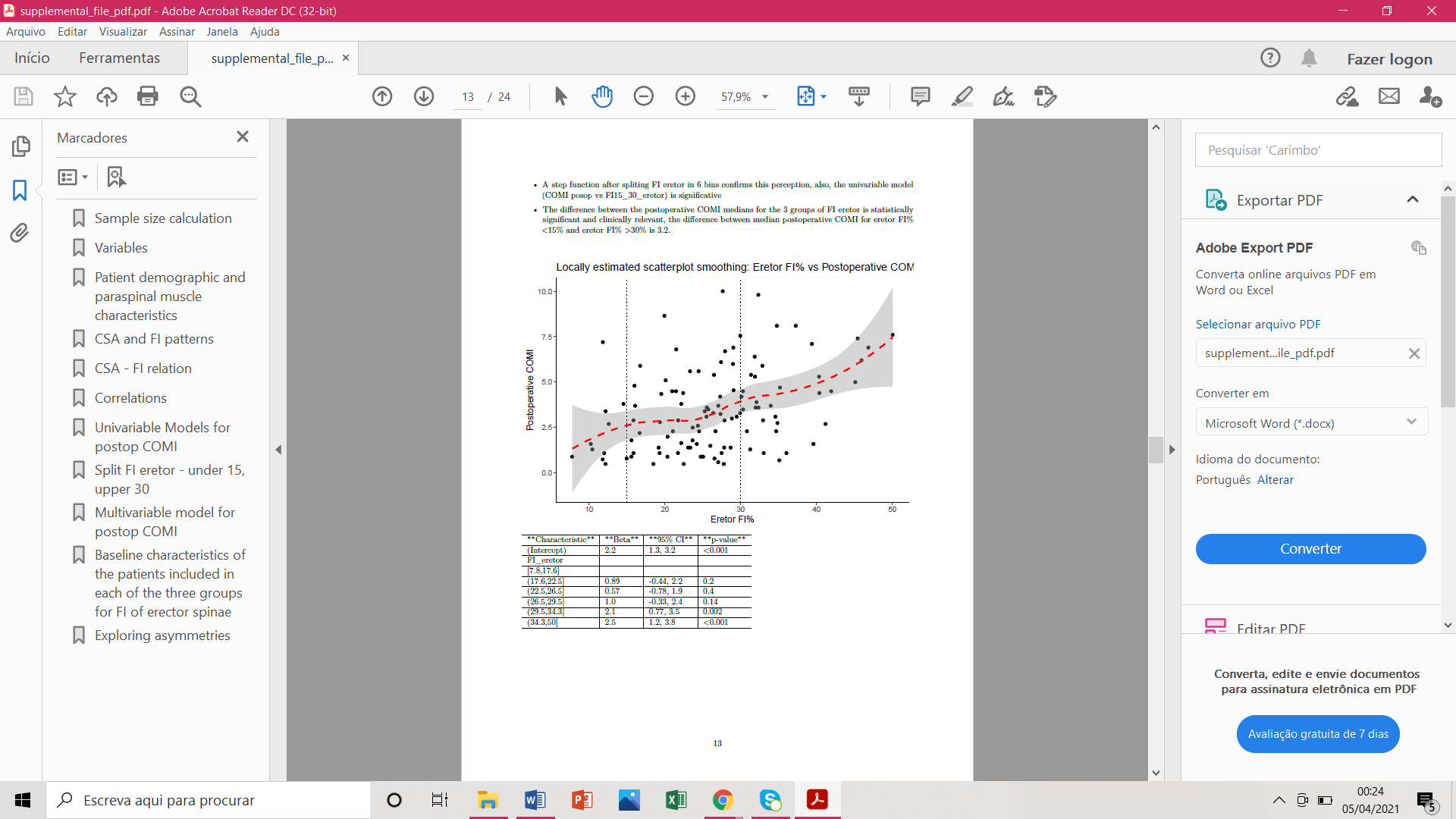


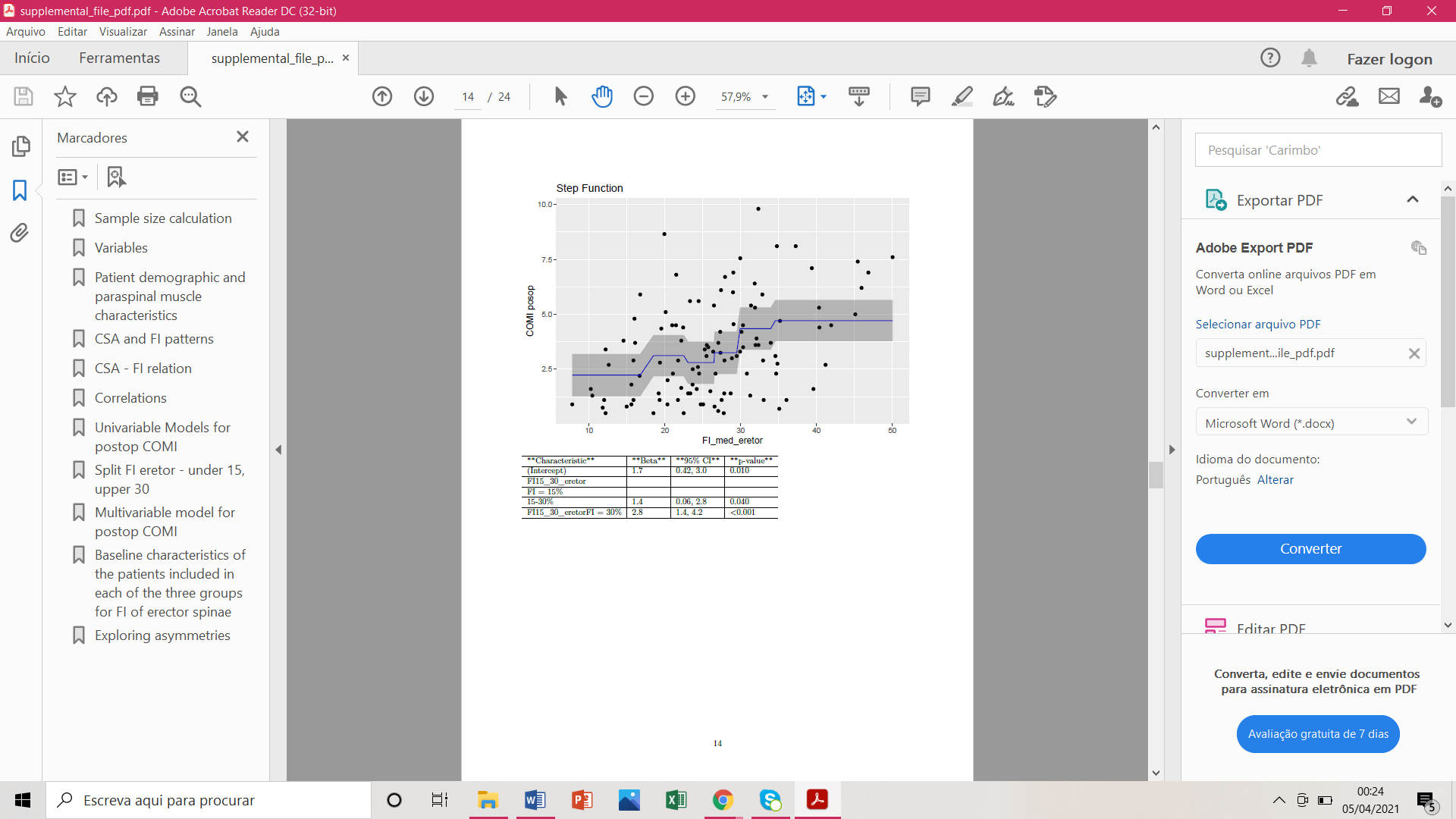


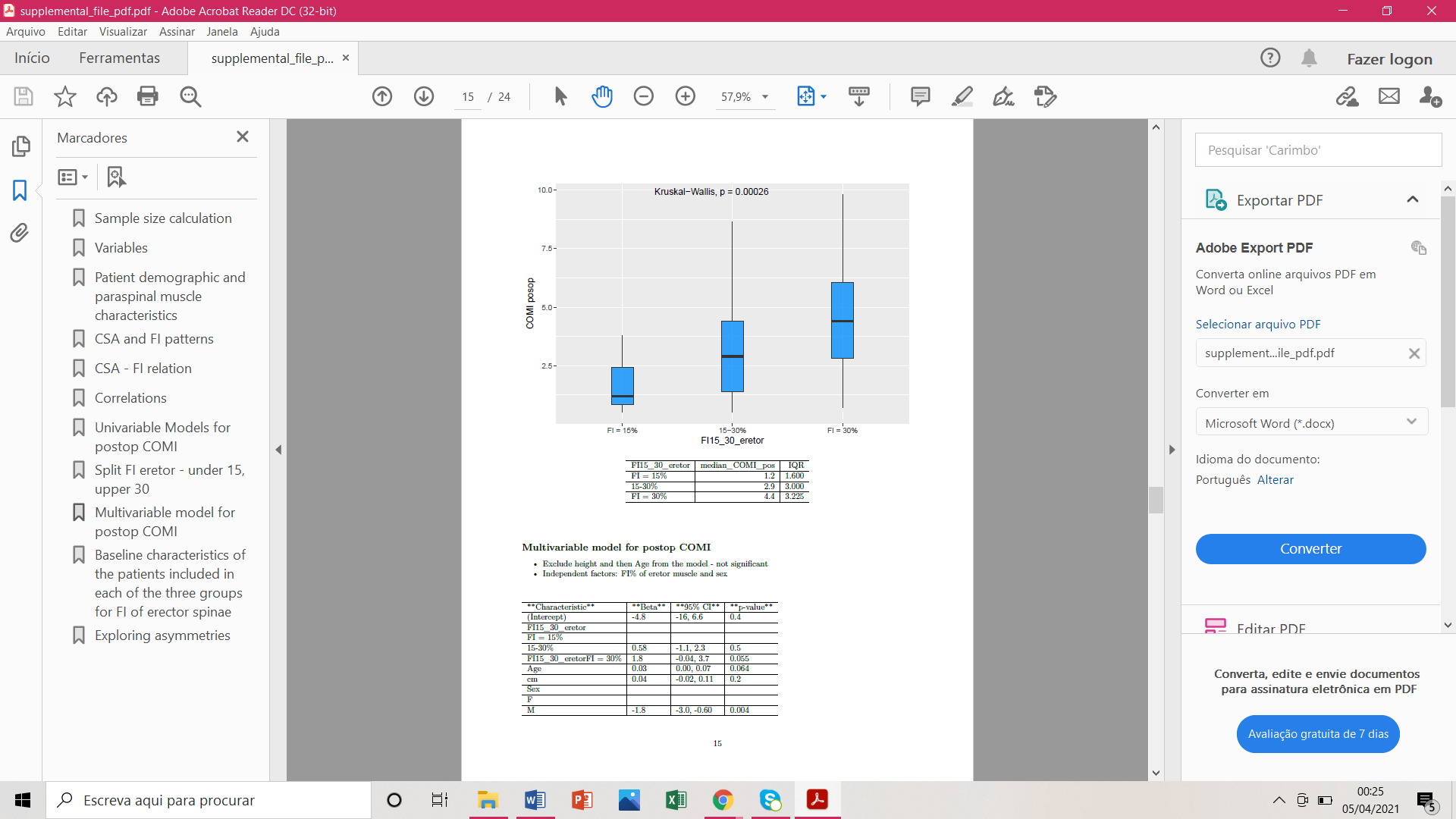


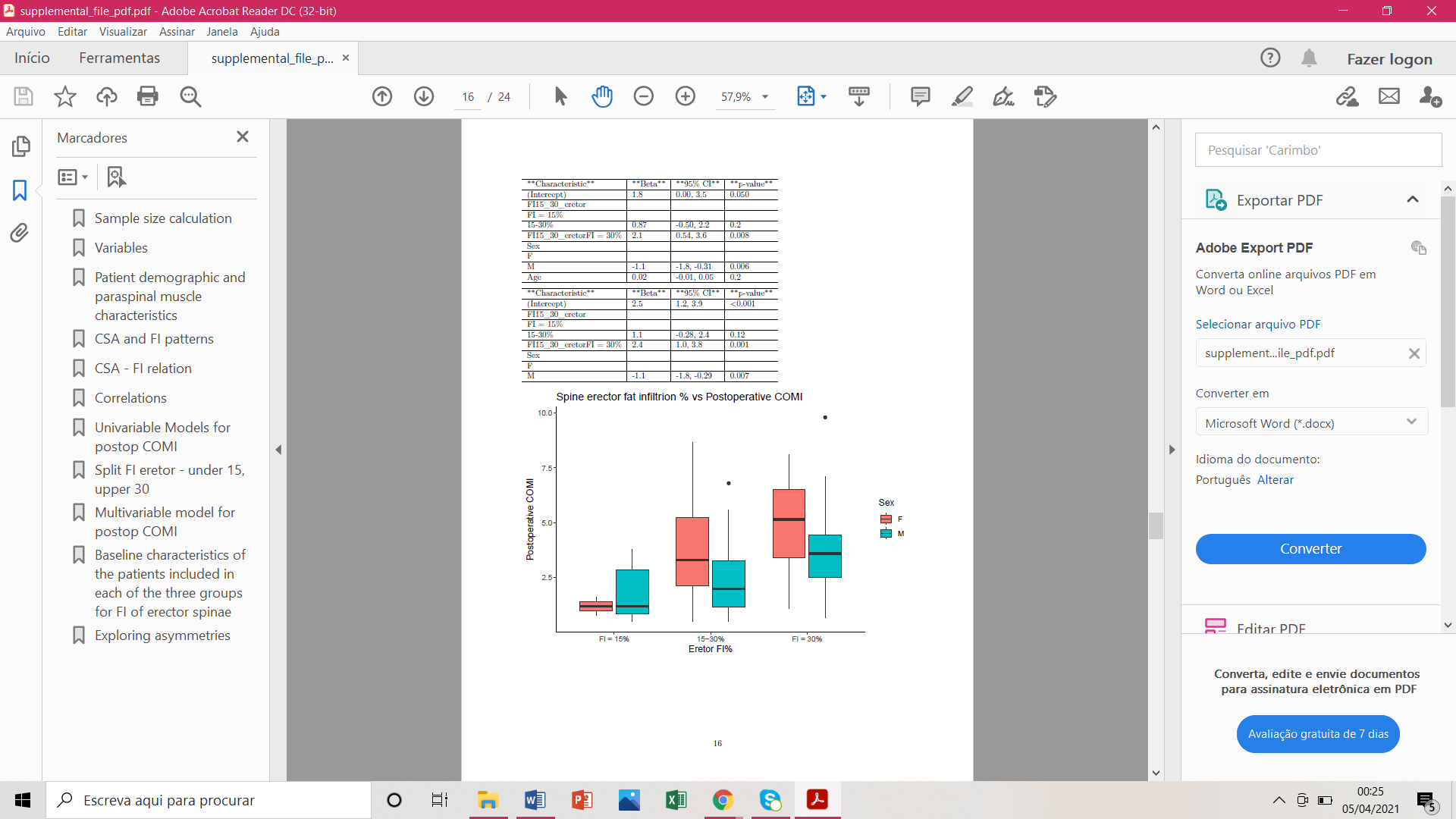


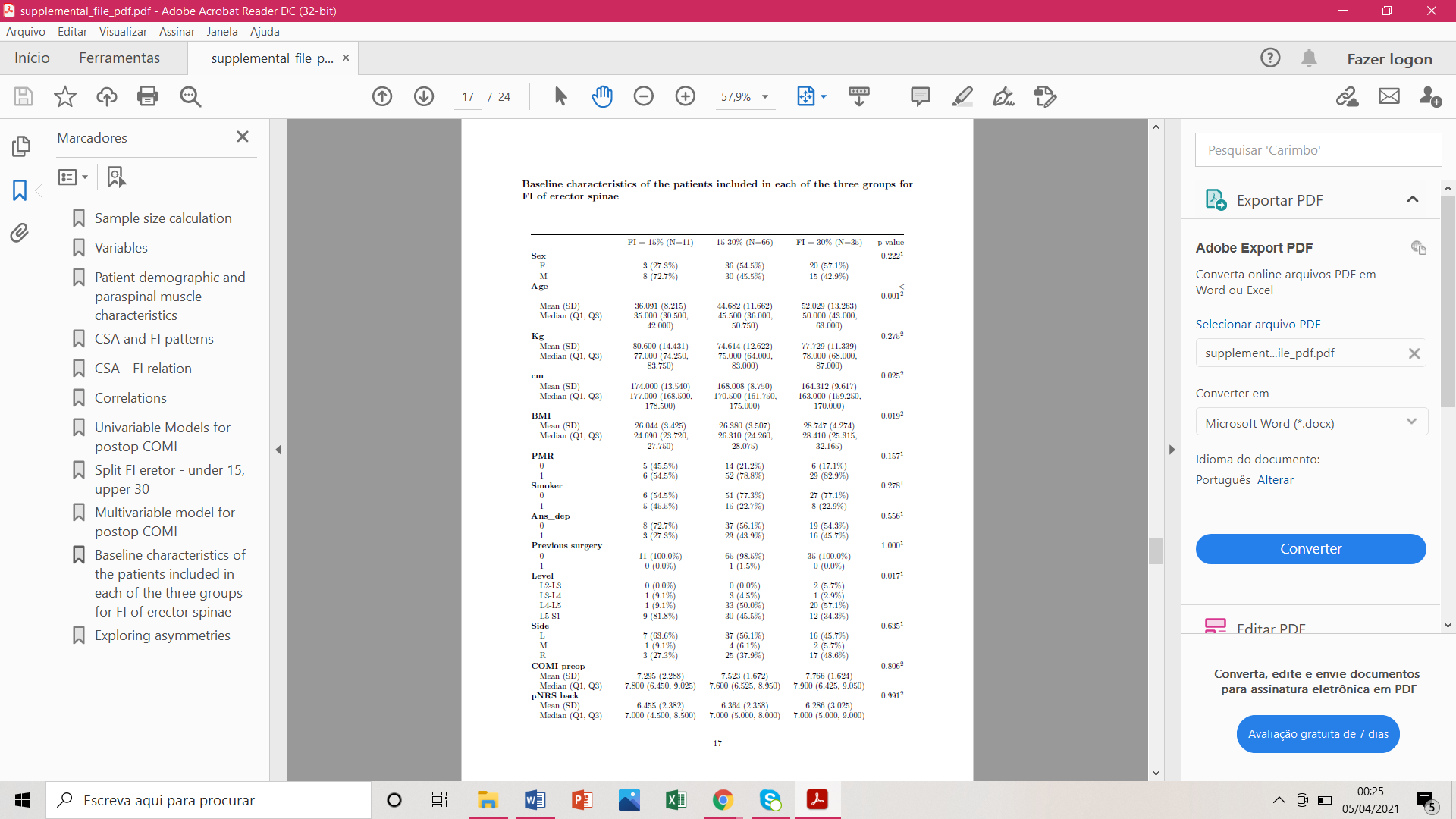


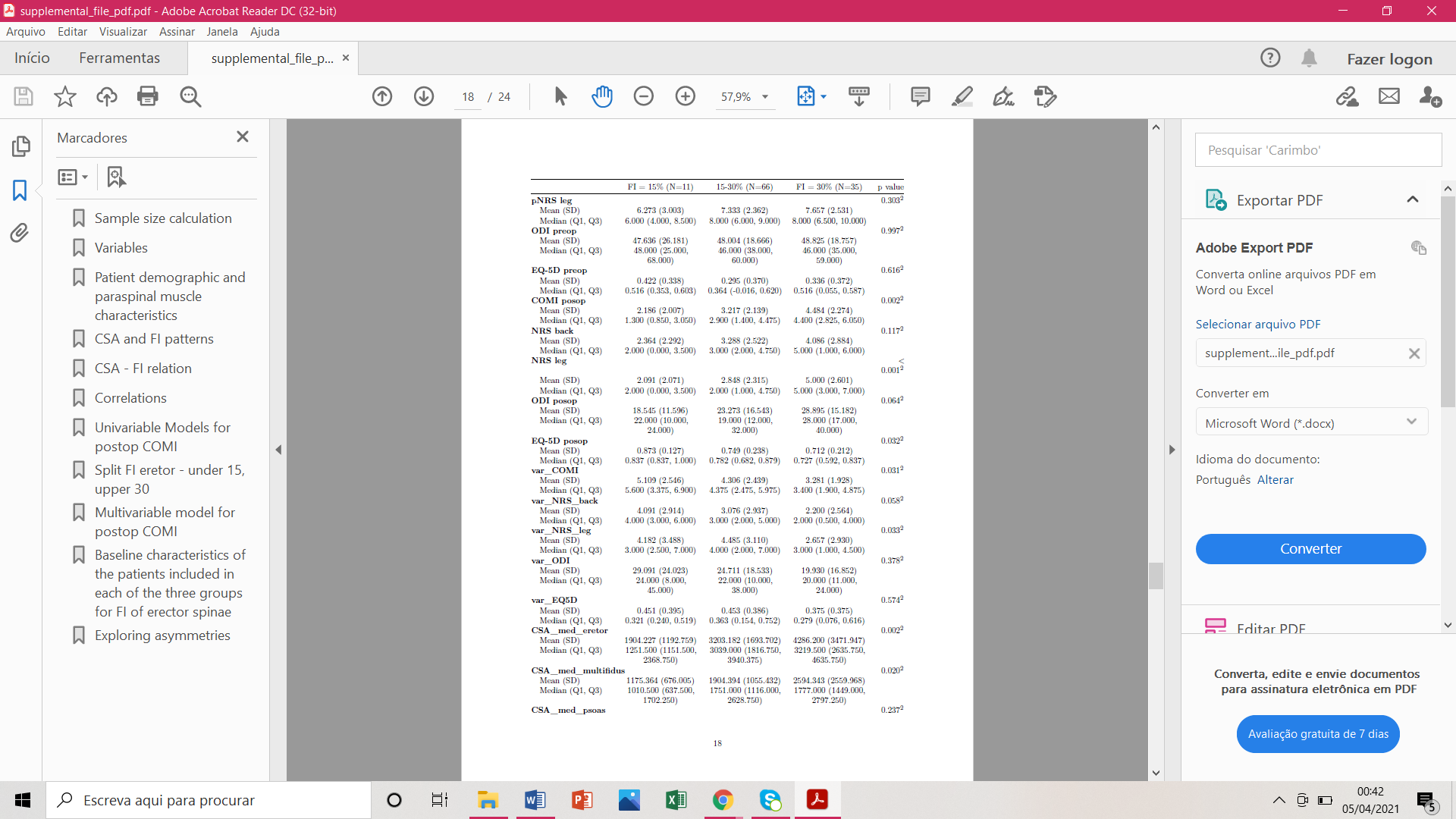


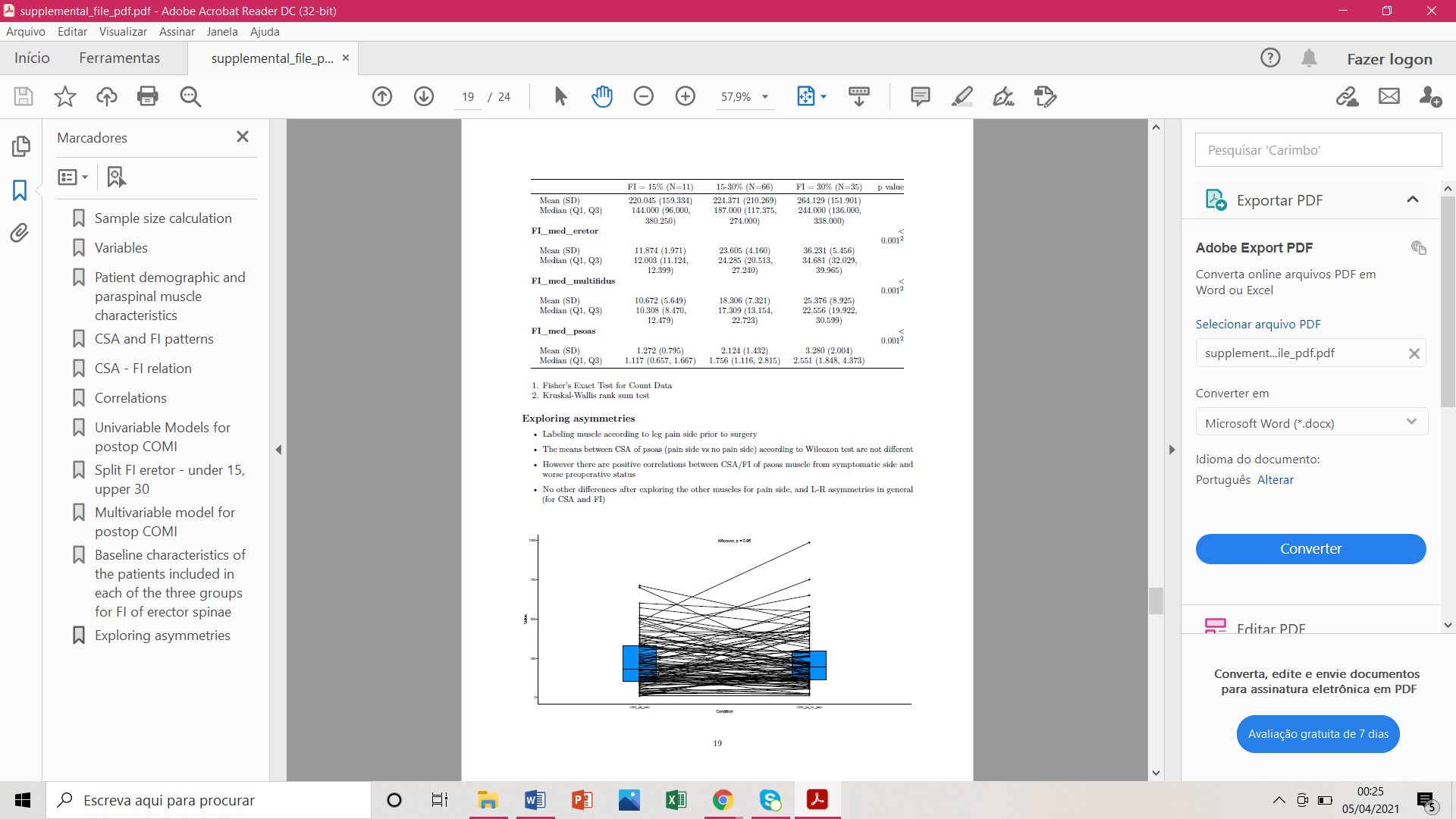


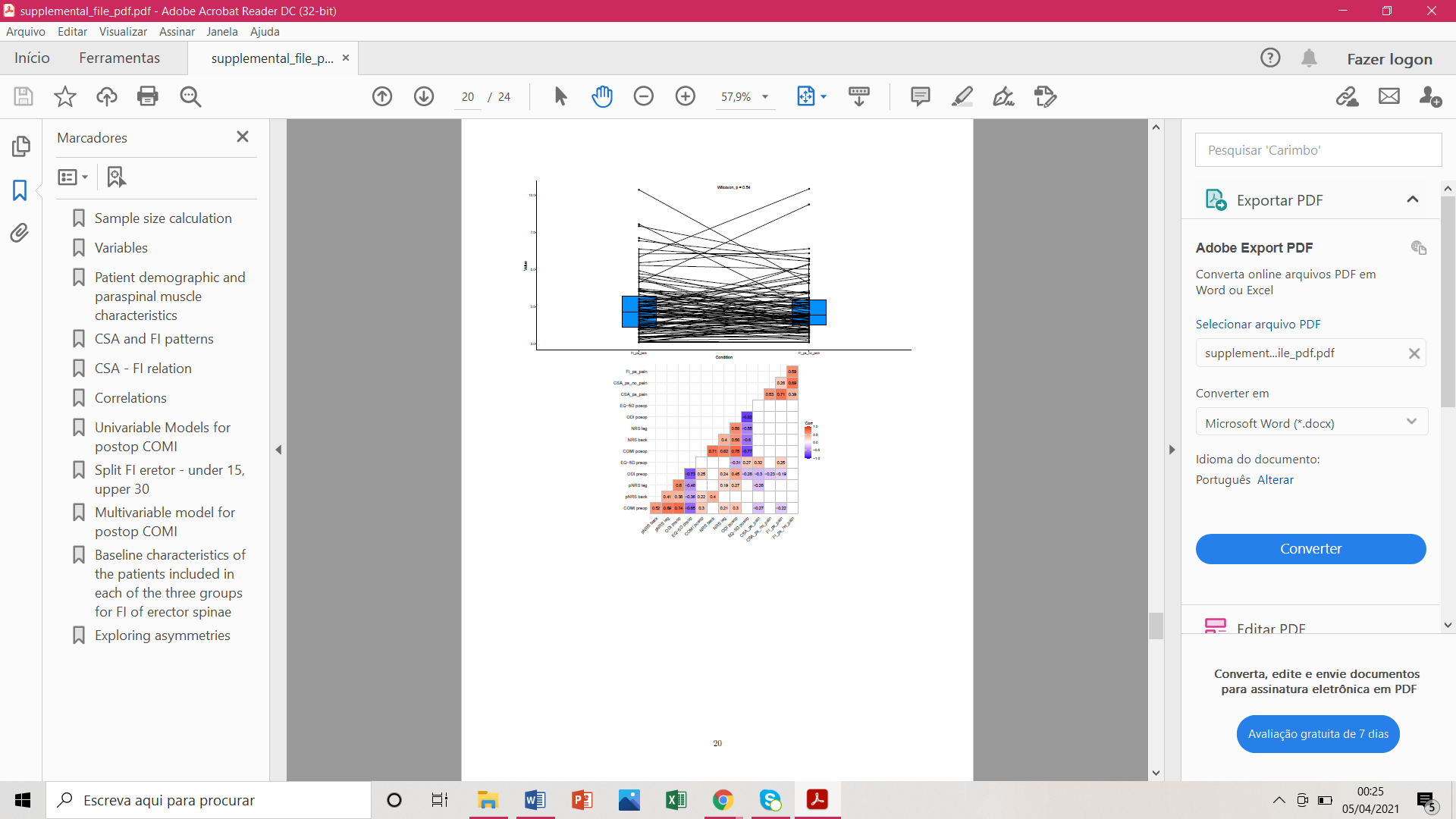


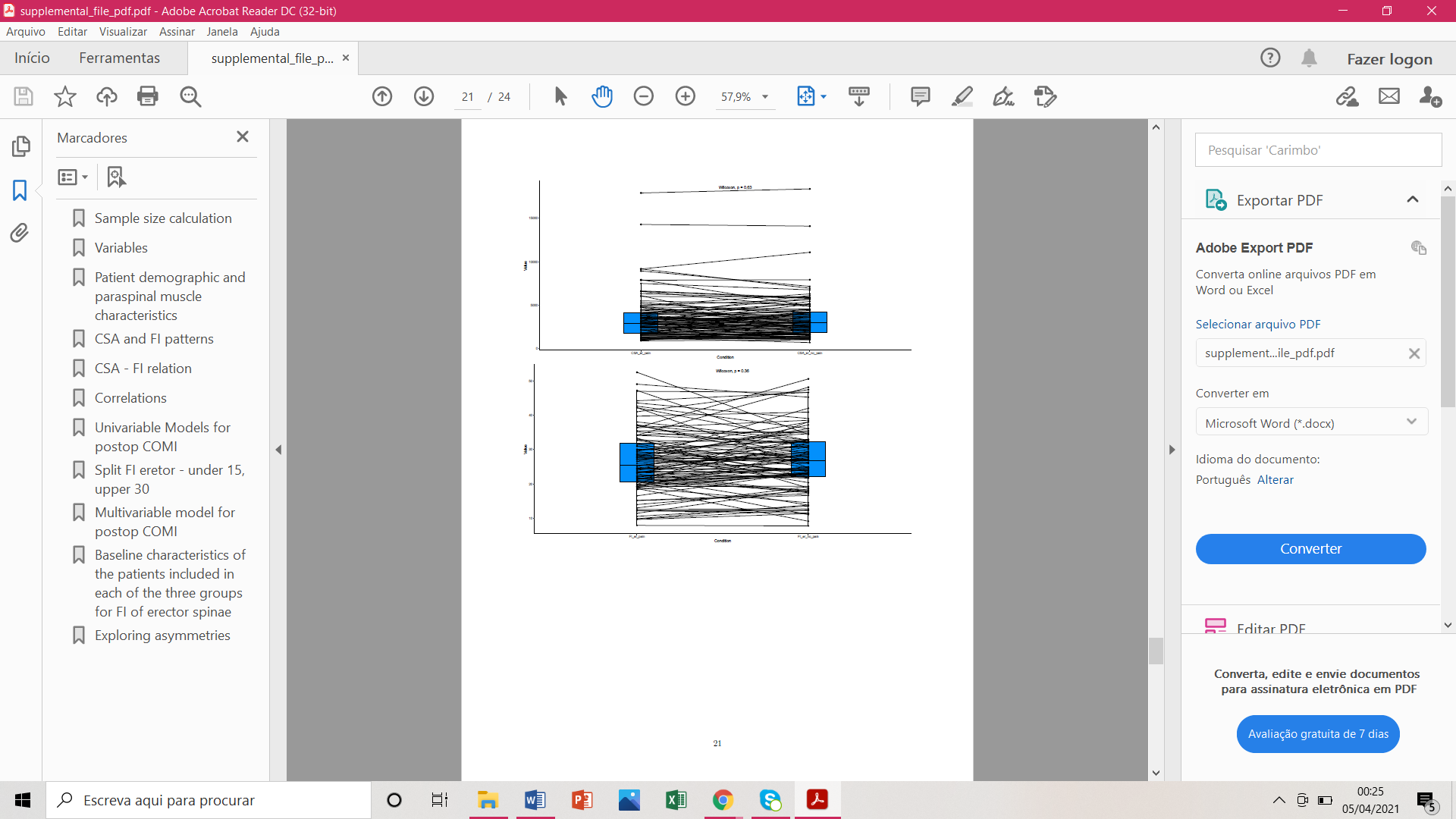


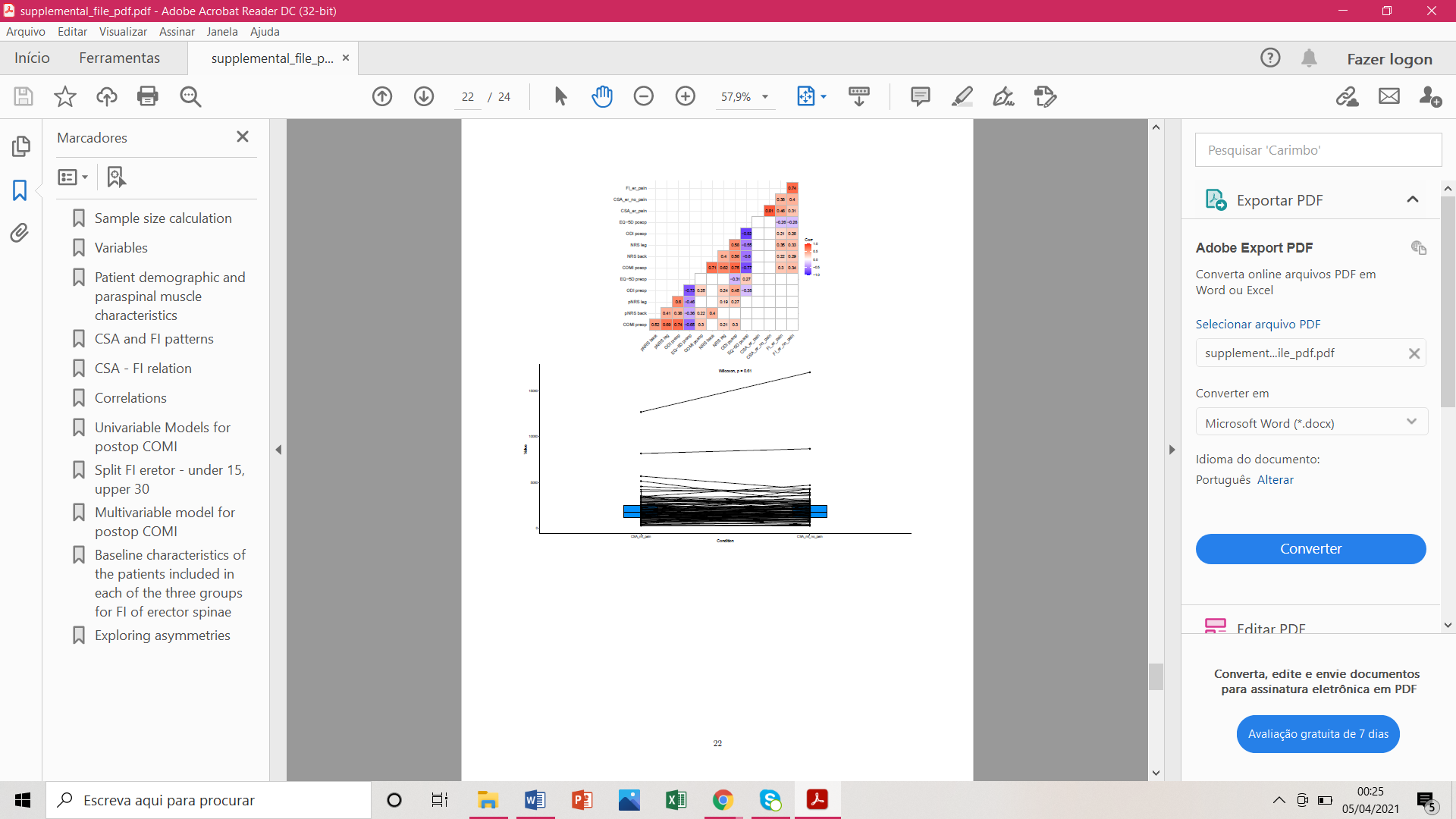


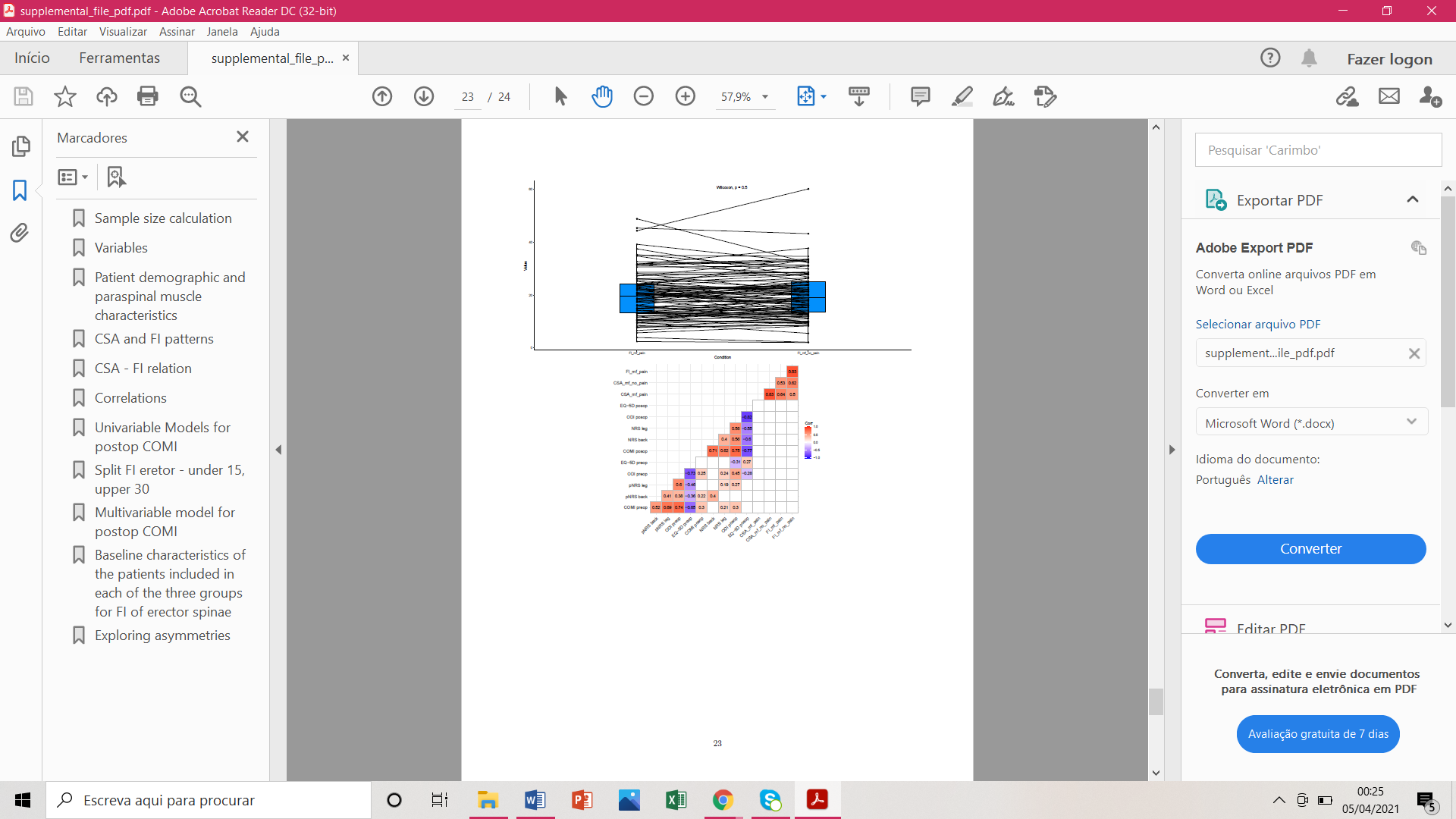


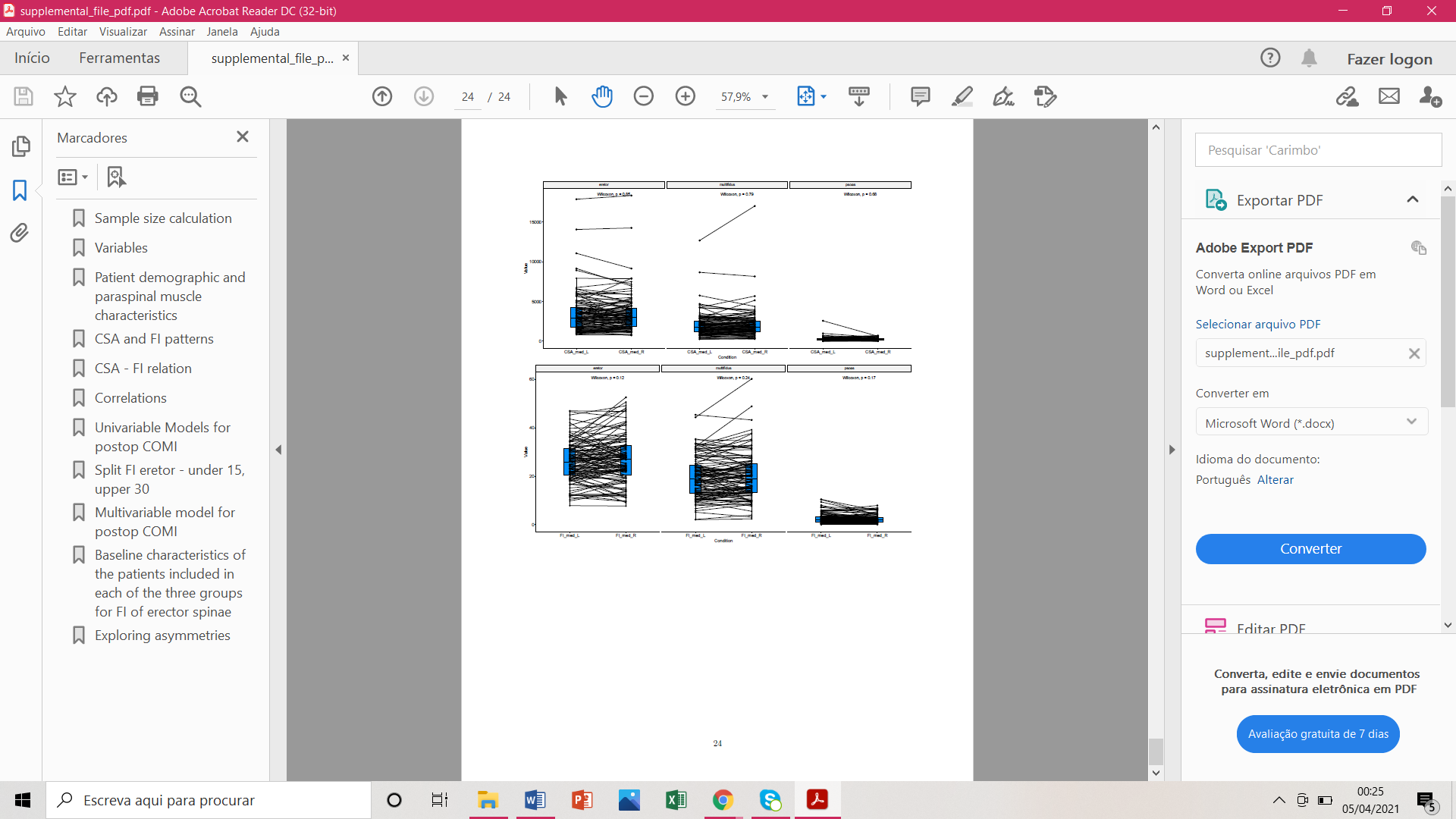

Supplement: Multimedia component 1 [file mmc1.docx]
